# Mutational Spectrum Analysis of Seven Genes Associated with Thyroid Dyshormonogenesis

**DOI:** 10.1155/2018/8986475

**Published:** 2018-08-02

**Authors:** Xi Chen, Xiaohong Kong, Jie Zhu, Tingting Zhang, Yanwei Li, Guifeng Ding, Huijuan Wang

**Affiliations:** ^1^Center for Genetic & Metabolic Disorders, Maternal and Child Health Care Hospital of Xinjiang Uygur Autonomous Region, Urumqi, China; ^2^The National Engineering Research Center for Miniaturized Detection Systems, College of Life Science, Northwest University, Xi'an, China

## Abstract

**Objective:**

Thyroid dyshormonogenesis (DH) is a genetically heterogeneous inherited disorder caused by thyroid hormone synthesis abnormalities. This study aims at comprehensively characterizing the mutation spectrum in Chinese patients with DH.

**Subjects and Methods:**

We utilized next-generation sequencing to screen for mutations in seven DH-associated genes (TPO, DUOX2, TG, DUOXA2, SLC26A4, SLC5A5, and IYD) in 21 Chinese Han patients with DH from Xinjiang Province.

**Results:**

Twenty-eight rare nonpolymorphic variants were found in 19 patients (90.5%), including 19, 5, 3, and 1 variants in DUOX2, TG, DUOXA2, and SLC26A4, respectively. Thirteen (62%) patients carried monogenic mutations, and six (28.5%) carried oligogenic mutations. Fifteen (71%) patients carried 2 or more DUOX2 (14) or DUOXA2 (1) variants. The genetic basis of DH in nine (43%) patients harboring biallelic or triallelic pathogenic variants was resolved. Seventeen patients (81%) carried DUOX2 mutations, most commonly p.R1110Q or p.K530X. No correlations were found between DUOX2 mutation types or numbers and clinical phenotypes.

**Conclusions:**

DUOX2 mutations were the most predominant genetic alterations of DH in the study cohort. Oligogenicity may explain the genetic basis of disease in many DH patients. Functional studies and further clinical studies with larger DH patient cohorts are needed to validate the roles of the mutations identified in this study.

## 1. Introduction

Congenital hypothyroidism (CH) is one of the most common and preventable endocrine disorders worldwide and affects 1 in 2000–4000 newborns [1]. Approximately 15–20% of CH cases result from thyroid dyshormonogenesis (DH) caused by mutations in thyroid hormone synthesis pathway genes, including those involved in hormone precursor production (thyroglobulin (TG)) [2], iodine transportation across the basal membrane (solute carrier family 5 member 5 (SLC5A5) [3], solute carrier family 26 member 4 (SLC26A4) [4]), thyroglobulin modification (thyroid peroxidase (TPO) [5], dual oxidase 2 gene (DUOX2) [6], and dual oxidase maturation factor 2 (DUOXA2) [7]), and iodine recycling in the thyroid (iodotyrosine deiodinase (IYD)) [8]. DH is generally clinically characterized by goiters and exhibits autosomal recessive inheritance [1, 9, 10].

Although hundreds of DH-related genetic mutations have been identified, the molecular basis for DH remains poorly understood. While DH is currently considered a monogenic disease, cases carrying digenic or oligogenic mutations have been reported [11–17]. The roles of oligogenicity in disease development and CH phenotypes must be clarified. Additionally, neither the mutational spectrum of DH-related genes nor DH phenotype-genotype correlations have been fully established. Patients with the same mutations in DUOX2, TG, or TPO demonstrate a broad spectrum of clinical phenotypes ranging from mild to severe CH or from transient to permanent hypothyroidism [12, 14, 18–22]. Current studies mainly focus on identifying mutations in common DH-related genes, such as TPO, DUOX2, and TG [8–20, 23, 24], and mutation incidences of other DH-related genes in CH patients are seldom reported. These rarely studied genes may work in concert with common genes to contribute to varied and complex phenotypes.

Wide utilization of next-generation sequencing (NGS) in clinical samples has identified mutations related to genetic disorders, but interpretation of sequence variants can be challenging. The pathogenic and functional roles of many identified variants remain unclear or controversial. Additionally, it appears that the genetic basis of DH is population-specific. Some studies suggest that TPO mutation is the major cause of DH in Caucasians [21, 22, 24], while in Asian populations, such as Japanese, Koreans, and Chinese, DUOX2 is the most common gene associated with DH [14, 18, 25, 26]. However, other studies gave different conclusions [27, 28]. Additional population-based studies are necessary to improve understanding of DH genetic heterogeneity in different populations.

The ethnically diverse Xinjiang Uygur Autonomous Region is located in the northwest border of China. CH incidence in this region is 1/1468, which is higher than the national average (1/3009) [29]. The Han nationality is the second largest ethnic group in Xinjiang, representing 40% of the total population [30]. Limited information is available regarding the CH mutation spectrum and genetic heterogeneity in Xinjiang populations. Here, we screened seven DH-related genes (TPO, DUOX2, DUOXA2, TG, SLC5A5, SLC26A4, and IYD) in 21 DH Han Chinese patients from Xinjiang using NGS.

## 2. Subjects and Methods

### 2.1. Subjects

Recruited patients were diagnosed and followed up at Urumqi Maternal and Child Health Care Hospital, Xinjiang, China, from October 2015 to May 2016. All patients underwent neonate thyroid screening for CH 2 h to 7 d after birth. Heel-puncture blood samples were collected on a filter paper to determine thyroid-stimulating hormone (TSH) levels using time-resolved fluorescence assay (Perkin Elmer, Waltham, MA, USA). Newborns with TSH levels > 8 *μ*IU/ml were reexamined to determine serum TSH and FT4 levels via electrochemiluminescence assay (Cobas e601, Roche Diagnostics, Indianapolis, IN, USA). CH diagnosis was based on elevated serum TSH (>10 *μ*IU/ml) and decreased FT4 levels (<0.93 ng/dl). L-T4 (levothyroxine) treatment was started for patients with serum TSH levels persistently >10 mU/l. Patients were classified as having permanent or transient CH based on the results of thyroid function tests after temporary withdrawal of L-thyroxine therapy at approximately three years of age. Patients with elevated TSH (TSH > 5 *μ*IU/ml) and decreased FT4 levels (FT4 < 0.93 ng/dl) at this time were considered to have permanent CH. Thyroid ultrasonography and ^99m^Tc scintigraphy were performed during the neonatal period prior to treatment. Patients with in situ normal-sized or enlarged (thyroid width ≥ −2 SD) thyroid gland were considered to have dyshormonogenesis, and thyroids ≥ + 2 SD in size were defined as goiters [31]. Within one year of age and again around two years of age, an intellectual assessment was performed for each patient using the 0–6-year-old pediatric neuropsychological development examination table provided by the Capital Institute of Pediatrics, China [32].

CH can be categorized as severe, moderate, or mild based on serum FT4 levels at diagnosis of <0.31, 0.31 to <0.62, or 0.62 to 0.93 ng/dl, respectively [23]. Based on these criteria, 21 cases were recruited for NGS analysis of seven known CH-related genes, and 32 cases were recruited as the validation cohort to verify the variants identified in the present study. Additionally, 100 infants with normal FT4 and TSH levels underwent neonate thyroid screening and were enrolled as a normal control group. All control subjects were Han Chinese from Xinjiang, including 58 males and 42 females, with a mean age of 29 days (range, 20–55 days). This study was approved by the Medical Ethics Committee of Urumqi Maternal and Child Health Care Hospital. All methods were performed in accordance with approved guidelines. Informed consent was obtained from all subjects or all parents.

To determine genetic mutations, peripheral blood samples (2–5 ml) were obtained from all subjects and transferred to the National Engineering Research Center for Miniaturized Detection Systems for molecular analysis.

### 2.2. DNA Extraction and Next-Generation Sequencing

Genomic DNA was manually extracted from peripheral blood using the Whole Blood Genomic DNA Isolation Kit (GoldMag, Xi'an, Shannxi, China). Isolated DNA was qualitatively and quantitatively analyzed via the Quant-iT™ dsDNA HS Assay (Invitrogen, Carlsbad, CA, USA) and Nanodrop spectrophotometer (Thermo Fisher Scientific, Wilmington, DE, USA), respectively.

Patients were genetically screened using a customized Ampliseq panel that included seven DH-associated genes (TPO, TG, DUOX2, DUOXA2, SLC5A5, SLC26A4, and IYD). Primers for the customized panel were designed using Ion AmpliSeq™ Designer (https://www.ampliseq.com/login/login.action) to cover coding exons and 20 flanking base pairs of the splice junctions surrounding exons of targeted genes. The Ampliseq design resulted in a total of 174 amplicons per sequencing run. Amplicon lengths ranged from 125–374 bp (median, 368 bp) (Table S1).

The library was prepared using the Ion AmpliSeq Library Kit 2.0 (Life Technologies, Carlsbad, CA, USA) with 10 ng of each genomic DNA sample and the Ion Xpress™ Barcode Adapter 1–16 Kit (Life Technologies, Carlsbad, CA, USA). Amplicon libraries were quantified using the 7500 Real-Time PCR System (Applied Biosystems, Foster City, CA, USA) prior to pooling into a collective template for subsequent processing.

Template preparation was performed using the Ion OneTouch™ 2.0 System and Ion OneTouch Enrichment System (Life Technologies). Amplicons were clonally amplified on ion sphere particles via emulsion PCR and then enriched with Ion PGM™ Enrichment Beads (Life Technologies). Finally, sequencing was performed using an Ion Torrent Personal Genome Machine (PGM) with the Ion PGM 200 Sequencing Kit and Ion 316™ Chip (Life Technologies) according to established procedures.

### 2.3. Variant Detection and Prioritization

Targeted sequencing data were analyzed via Torrent Suite software (v5.0.4; Life Technologies). Each read was aligned to the hg19 human reference genome to detect variants. Called variants were functionally annotated using Ion Reporter software (https://ionreporter.lifetechnologies.com/ir/secure/home.html) and the ANNOVAR package (http://wannovar.wglab.org/). Variants were further filtered using the dbSNP database (https://www.ncbi.nlm.nih.gov/projects/SNP/), 1000 Genomes Project (https://ftp.ncbi.nih.gov/), Exome Sequencing Project (http://evs.gs.washington.edu/EVS/), and Exome Aggregation Consortium (ExAC, http://exac.broadinstitute.org/). Variants with minor allele frequencies > 0.01 and synonymous variants were excluded. Additionally, variants associated with CH in the published literature or by the Human Gene Mutation Database (HGMD® Professional 2017.2, https://portal.biobase-international.com/hgmd/pro/start.php) were included in the analysis. All variants filtered by the above criteria were verified by Sanger sequencing with ABI3500 xL Dx (Applied Biosystems) (Table S2). Finally, frequencies of validated variants were determined in the normal control and the validation patient cohort by Sanger sequencing.

### 2.4. Pathogenicity Assessment

All variants were classified following ACMG/AMP standards and guidelines [33]. Six major evidence categories were established: (1) population data from the 1000 Genomes Project, ExAC, and our local normal control; (2) computational prediction data, wherein the possible functional significance of missense or indel variants was assessed using five in silico tools, including Sorting Intolerant from Tolerant (SIFT, http://provean.jcvi.org/genome_submit_2.php), Polymorphism Phenotyping v2 (PolyPhen-2, http://genetics.bwh.harvard.edu/pph2/index.shtml), MutationTaster (http://www.mutationtaster.org/), Functional Analysis through Hidden Markov Models v2.3 (FATHMM, http://fathmm.biocompute.org.uk/), and Mendelian Clinically Applicable Pathogenicity (M-CAP, http://bejerano.stanford.edu/MCAP/), and the deleterious effect of the splicing mutation on RNA splicing was predicted using MaxEntScan (http://genes.mit.edu/burgelab/maxent/Xmaxentscan_scoreseq.html), Berkeley Drosophila Genome Project (BDGP, http://www.fruitfly.org/seq_tools/splice.html), and NetGene2 (http://www.cbs.dtu.dk/services/NetGene2/); (3) mutation types, predicted null variants in a gene where loss of function (LOF) is a known mechanism of disease; (4) evolutionary conservation analysis of variants, which was performed by DNAMAN 8 [34], and protein domain and structure from UniProt Knowledgebase (https://www.uniprot.org/); (5) experimental functional data from published literature; and (6) family segregation analysis data from the present study or the published literature.

## 3. Results

### 3.1. Patient Demographics and Clinical Characteristics

This study included 21 Chinese Han patients with DH from unrelated families for NGS analysis (Table 1). Patients included 13 female and 8 male subjects ranging in age from 1 year and 2 months to 5 years and 11 months. Three cases with CH and goiters (thyroid widths > 1.9 cm) were diagnosed via ^99m^Tc thyroid scan. Five cases demonstrated low neuropsychological development around the age of two. Of the five cases older than three years and who underwent therapy withdrawal, one had temporary CH and four had permanent CH. Based on FT4 levels at diagnosis, 7, 7, and 7 patients were biologically classified as mild, moderate, and severe CH. Additional 32 DH patients of Han nationality were recruited as a validation cohort, which consisted of 20 females and 12 males, ranging in age from 9 months to 9 years and 8 months (Table S5). Parental samples were obtained in five cases (patients 3, 5, 7, 15, and 19) for family segregation analysis.

### 3.2. Sequencing Data Analysis

NGS of the seven target genes was performed for the 21 CH patients. The total number of mapped reads for individual samples ranged from 89,050 to 942,537 (median, 187,649; *n* = 21). The median percentage of on-target sequences in each sample was 99%, with an average base coverage depth ranging from 371× to 4207× for individual samples. The average total coverage of all targeted bases was 98.62% at 20×, 94.55% at 100×, and 74.91% at 500×. The coverage was uniform across all samples. On average, 91% of called bases had a quality score ≥ Q20 (Table S3).

### 3.3. Variant Detection

Overall, 204 single-nucleotide variants (SNVs) and 8 indels were called in the 21 patients. The number of variants ranged from 54 to 104 per patient. Variant annotation indicated that 156 (76.5%) of the variants were predicted to be noncoding or synonymous, whereas 48 (23.5%) were nonsynonymous and insertion or deletion variants that lead to alterations in one or more amino acids.

**Figure 1:**
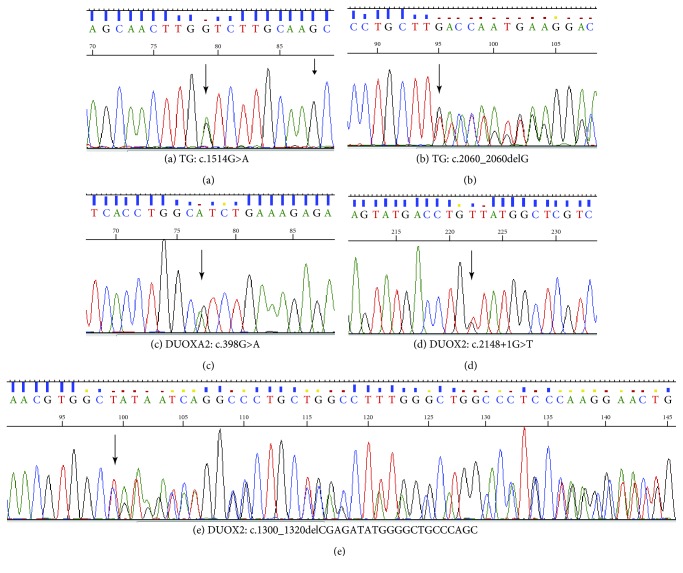
Sequencing chromatograms of five novel variants detected in this study: (a) c.1514G>A in TG; (b) c.2060_2060delG in TG; (c) c.389G>A in DUOXA2; (d) c.2148+1G>T in DUOX2; (e) c.1300_1320delCGAGATATGGGGCTGCCCAGC in DUOX2.

**Figure 2:**
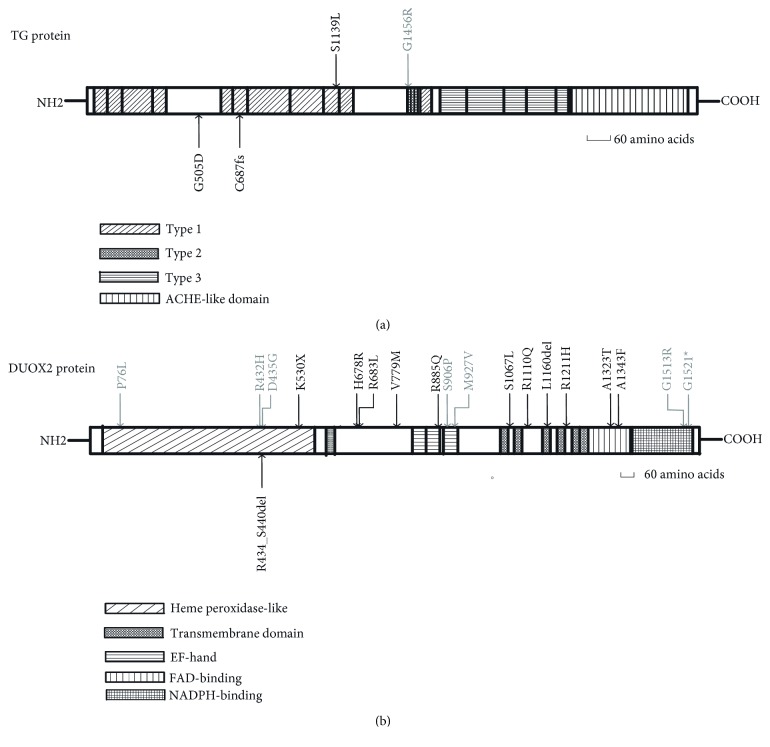
Location of detected missense or indel variants in TG and DUOX2 proteins. (a) Four mutations in TG. The repetitive units (types 1, 2, and 3) and ACHE-like domain are represented by boxes [19]. (b) Nineteen variants, including two functional SNPs, in DUOX2. The relative positions of the transmembrane domain, heme peroxidase-like domain, calcium-binding (EF-hand), flavine adenine dinucleotide- (FAD-) binding, and reduced nicotinamide adenine dinucleotide phosphate- (NADPH-) binding motifs are indicated [46]. Labeled variants (top) represent previously reported variants. Variants previously reported in the literature are shown in black, and those reported only in public population databases are shown in gray. Labeled variants (bottom) are novel variants identified in the present study.

After functional filtering, a total of 28 rare nonpolymorphic variants in 19 patients (90.5%) were identified, including 4 indels, 3 splice variants, and 21 single-nucleotide substitutions (Table 2). All were absent in local control samples. Among these variants, five were identified for the first time in this study (Figure 1), 14 had been reported in the published literature and HGMD (HGMD Professional 2017.2), and nine were previously reported in dbSNP, ExAC, and/or the 1000 Genomes Project database, although phenotypic data were lacking. Two novel variants were found in DUOX2, including an indel (c.1300_1320delCGAGATATGGGGCTGCCCAGC) and a splice variant (IVS17+1G>T). The former variant caused the deletion of seven amino acids in exon 12 (p.R434_S440del). These seven amino acids are located in the peroxidase- (PO-) like domain and are conserved among DUOX2 orthologs (Figure 2 and Figure S1). The latter variant likely resulted in aberrant splicing of the transcript. Two novel variants were identified in TG, including one frameshift mutation (c.2060_2060delG, p.C687LfsX34) and one missense mutation (c.1514G>A, p.G505D). A novel missense mutation was found in DUOXA2 (c.398G>A, p.R133H).

Besides 28 rare nonpolymorphic variants, two polymorphic variants in DUOX2, p.H678R and p.S1067L, were commonly identified with frequencies of 0.19 and 0.286, respectively, which were higher than those in the controls (0.19 versus 0.092, OR (odds ratio) = 2.327, *P* = 0.097; 0.286 versus 0.085, OR = 4.306, *P* = 0.001). These two variants were, respectively, reported as a disease-associated polymorphism and a likely disease-caused mutation in HGMD. In a validation cohort including 32 Chinese Han DH patients from Xinjiang, p.H678R and p.S1067L were also commonly detected and were associated with DH risk (p.H678R: OR = 2.521, *P* = 0.025; p.S1067L: OR = 3.894, *P* < 0.000) (Table S5). These two SNPs often cooccurred in patients with DUOX2 mutations. Linkage disequilibrium analysis showed that these two variants were highly linked in both the studied patient cohort (D′ = 1, *R* ^2^ = 0.58) and the validation cohort (D′ = 1, *R* ^2^ = 0.70) but were weakly linked in controls (D′ = 0.22, *R* ^2^ = 0.04).

Among the seven analyzed candidate genes, DUOX2 mutations were the most frequent genetic alterations in DH. 19/28 rare variants (68%) were in DUOX2, and approximately 81% (17/21) of patients had DUOX2 mutations. p.R1110Q was the most common DUOX2 mutation in the patient cohort, with an allelic frequency of 0.143. Including the validation cohort, p.K530X, IVS28+1G>T, p.R885Q, p.L1343F, and p.R683L were also common in Xinjiang DH patients. TG mutations were the second most prevalent genetic alterations in DH: five different heterozygous variants were found in 5/21 patients (23.8%), and these often cooccurred with DUOX2 or DUOXA2 mutations. DUOX2 and TG mutation locations varied in the corresponding proteins (Figure 2). Additionally, three DUOXA2 variants were found in 3/21 patients (14%), and a known heterozygous variant in SLC26A4 was found in one patient. No mutations in SLC5A5, TPO, or IYD gene exons were found.

Most of the variants presented as heterozygous in patients. Only three variants were homozygous in three patients: (1) DUOX2: c.2779A>G (p.M927V) in one patient, (2) DUOX2:c.3329G>A (p.R1110Q) in one patient, and (3) DUOXA2: c.413dupA (p.Y138X) in one patient.

### 3.4. Pathogenicity Assessment

The pathogenicities of detected variants were classified according to the American College of Medical Genetics and Genomics and the Association for Molecular Pathology (ACMG/AMP) standards and guidelines [33]. Of the 28 rare variants, eight were truncating or null variants, including four nonsense (DUOX2 gene: p.K530X and p.G1521X; DUOXA2 gene: p.Y246X and p.Y138X), three splicing (DUOX2 gene: IVS17+1G>T, IVS28+1G>T; TG: IVS10-1G>A), and one frameshift mutation (TG gene: p.C687LfsX34). All variants were located at highly conserved regions or critical functional domains and were predicted to be disease causing by computational software (Table 3 and Table S4, Figure 2 and Figure S1). These variants were classified as pathogenic, with the exception of one nonsense variant, DUOX2 c.4561G>T (p.G1521X). This variant was located in the last DUOX2 exon and resulted in a prestop codon in the last 50 amino acids of the NADPH-binding region. Although MutationTaster predicted that this mutation has a deleterious effect on protein function, evidence could not support its pathogenic status. Therefore, this mutation was classified as a variant of uncertain significance (VUS) (Figure 2, Table 3 and Table S4). Of the 20 missense or indel variants, four known variants (p.R885Q, p.S906P, p.R1110Q, and p.L1160del) and one novel variant (p.R434_S440del) in DUOX2 were classified as pathogenic or likely pathogenic, and sixteen were classified as VUS owing to lack of sufficient evidence to support their pathogenic or benign statuses (Table S4). The two DUOX2 polymorphic variants, p.S1067L and p.H678R, were classified as benign (Table S4).

### 3.5. Genotype and Phenotype Relationships

**Figure 3:**
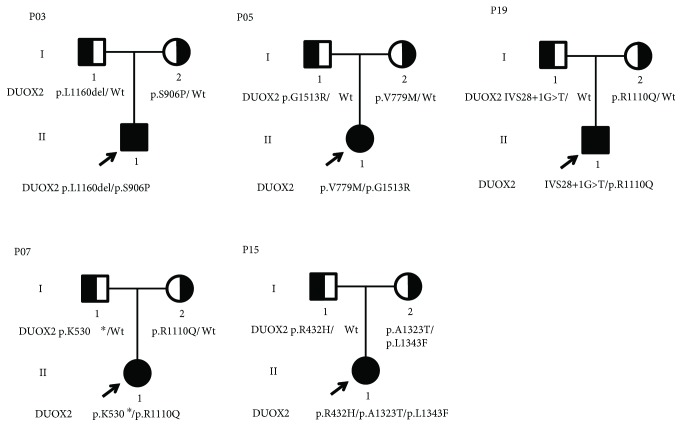
Genotypes and pedigrees of five DH patients. Arrow: proband; Roman numbers: generations; squares: males; circles: females; half-filled symbols: unaffected heterozygote individuals.

Except patients 4 and 21, all patients had one or more rare variants or alleles. Patient 21 carried no mutations but was compound for two heterozygous polymorphisms (p.H678R and p.S1067L). Nine patients (number 2, 3, 6, 7, 8, 10, 14, 18, and 19) carried homozygous or double heterozygous pathogenic variants in a single gene, including eight patients who carried DUOX2 mutations and one who carried DUOXA2 mutations, and their genetic basis was clarified. The pathogenicities of ten patients were ambiguous, due to the VUS they carried. Of these patients, one carried one heterozygous DUOX2 variant, three harbored two or three heterozygous variants in DUOX2, and six carried oligogenic mutations, including five cases comprising DUOX2 mutations plus a heterozygous mutation in TG or DUOXA2 and one case carrying a single heterozygous mutation each in DUOXA2, SLC26A4, and TG. With available parental DNA samples, identified variants carried by the five cases (patients 3, 5, 7, 15, and 19) were of either paternal or maternal origin, and none came from one single parent (Figure 3).

DUOX2 mutation numbers and types carried by patients were not correlated with CH clinical phenotypes, including disease severity, neuropsychological development, or prognosis. For example, patients 14 and 19 each carried one known truncating mutation (IVS28+1G>T) and a known inactivating mutation (p.R110Q or p.R885Q). One showed severe CH and low intelligence level, and the other showed mild CH and normal intelligence. Similarly, patients 8 and 10 both had a combination of a known truncating mutation (p.K530X) and a known inactivating mutation (p.R110Q or p.R885Q); one exhibited permanent CH and one showed transient hypothyroidism. Furthermore, patient 7 had exactly the same mutations as patient 8, and her prognosis was unknown. Unlike patient 8, who had a goiter, patient 7's thyroid size was normal. Moreover, numbers of detected variants differed among patients who shared the same phenotypes.

## 4. Discussion

Thyroid hormone biosynthesis defects are common causes of CH. Mutations in DH-associated genes, including TPO, TG, DUOX2, DUOXA2, SLC26A4, SCL5A5, and IYD, have been detected in numerous cases [9, 12, 18]. Although dual oxidase 1 (DUOX1) and dual oxidase maturation factor 1 (DUOXA1) have established roles in thyroid hormone production [35–37], relevant mutations associated with CH have not been found. Therefore, we designed a specific NGS panel to comprehensively identify pathological mutations in DH patients of Han nationality in the Xinjiang area. We found that nearly 85.7% (18/21) of patients screened in this study had two or more rare genetic variants or alleles. These results generally agreed with data reported for Japanese and Chinese DH cohorts and further support DH as a highly heritable recessive trait [15, 18].

Previous studies showed that DUOX2 mutation is highly prevalent in East Asians, such as Han Chinese [15, 26, 38–41], Japanese [18, 25], and Koreans [13, 14], and DUOX2 is the main gene responsible for DH. In agreement with previous reports, we identified DUOX2 as the leading genetic alteration of DH in our Xinjiang Han Chinese study population, with a detection rate of 81% (17/21 cases). Furthermore, 67% of patients (14/21) carried homozygous or compound heterozygous DUOX2 variants, which was similar to the rates reported in other Han Chinese populations [15, 40] but was higher than those (43%) reported in a Japanese DH patient cohort [18]. p.R1110Q was the most common mutation identified in our patient cohort, which differed from previous reports in Korean (p.G488R) [13, 14] and Japanese (p.R855Q) populations [11]. Additionally, p.K530X was the most common mutation identified in Chinese patients from southern or central China [15, 38–40].

Besides DUOX2, TG anomalies are another common cause of DH [19]. However, in the present study, four detected TG variants presented separately in four different patients with heterozygosity and always cooccurred with variants in DUOX2 or other DH-related genes, indicating that the contributions of TG mutations to DH in Xinjiang Han Chinese might be less important. More CH-associated DUOXA2 mutations were found recently [7, 42, 43]. Our study identified two known truncating variants, p.Y246X and p.Y138X, which cooccurred in a patient with permanent CH. A previous study first noted p.Y246X homozygosity in a patient with mild permanent CH and dyshormonogenic goiter [7], and compound heterozygosity with p.Y138X and p.Y246X was reported in another patient [43]. These cases were of Chinese origin, suggesting that p.Y246X and p.Y138X are specific pathogenic variants in Chinese populations. TPO mutations are more prevalent in DH patients of Caucasian origin than in the Chinese patients in the present study [21, 22, 24]. It appears that the genetic basis of DH differs according to patient ethnicity, although some studies gave different conclusions. Muzza et al. reported a high prevalence (37%) of DUOX2 mutation in a Caucasian CH cohort [27]. DH-related gene mutation spectrum discrepancies in different studies may be attributed to different sampling criteria, sample sizes, and/or mutation detection methodologies.

The pathogenicities of all detected variants were reassessed to further understand the DH mutation spectrum. Using the new and more stringent ACMG/AMP guidelines [33], we found that our classification of some known variants was inconsistent with that by HGMD or the published literature. For example, two known missense mutations, DUOX2 p.V779M and p.R1211H, are, respectively, annotated as possibly pathological and pathological in HGMD but are classified as VUS in the present study, due to the absence of functional data. In addition, two polymorphic variants, DUOX2 p.H678R and p.S1067L, were, respectively, annotated as a disease-associated polymorphism and a likely pathological variant in HGMD database. In the published literatures, the functional roles of these two variants and their correlations with DH are still disputed [11, 14, 18, 27, 44]. We detected these two variants at higher rates in patients than in healthy controls and found that they were associated with higher DH risk. Our findings were similar to those in Korean (p.H678R: 0.134 versus 0.055, *P* = 0.04) [14] and Japanese (p.H678R: 0.103 versus 0.035, *P* = 0.006, OR = 3.6; p.S1067L: 0.142 versus 0.058, *P* = 0.0017, OR = 2.67) populations [18]. Linkage disequilibrium analysis showed that these two variants were highly linked in CH patients but weakly linked in controls, and they often cooccurred with other DUOX2 mutations. Thus, we tended to conclude that these variants were disease-associated polymorphisms. They may not solely cause CH but could be used as CH predictors and may combine with other mutations or unidentified factors to induce CH.

Although biallelic and monogenic mutations are now considered as the most common causes of DH, concern has increased about the roles of oligogenic defects in CH pathogenesis and the CH phenotype. In this study, six patients (28.5%) carried variants in multiple DH-related genes. More cases with oligogenic mutations have been reported [11–17, 39, 45]. Two recently published studies which assessed multiple genes simultaneously [12, 17] have reported frequent oligogenic involvement (20–26.2%) in CH patients, although they assessed different ethnic populations. Oligogenicity may contribute to the varied phenotypes of CH patients, especially in association with known pathogenic DUOX2 mutations. However, due to small pedigree sizes and limited information about genotype-phenotype correlations, the relative etiological contribution of oligogenicity in CH is still uncertain.

As the predominant causes of DH, DUOX2 mutation genotype-phenotype relationships are greatly varied. Moreno et al. reported that permanent CH is associated with biallelic inactivating DUOX2 mutations and transient CH with monoallelic mutations [6], and some studies suggested DUOX2 mutations were often associated with mild to moderate phenotypes [11, 20, 25, 46]. However, subsequent studies showed that the permanent or transient nature of CH is not directly related to the number of inactivated DUOX2 alleles, and the link between DUOX2 genotype and CH phenotype remains unclear [14, 20, 26, 27, 40, 44, 47]. We found that DUOX2 mutation types or numbers did not directly correlate with disease severity (biologically classified via serum TG level at diagnosis), neurodevelopment, or prognosis. Therefore, the extremely complex relationship between DUOX2 genotypes and clinical phenotypes suggests that currently unidentified genetic/environmental factors may lead to the variety observed in the patient clinical manifestations [11, 18, 47].

In conclusion, this was the first reported mutation screening study for seven DH-related genes in DH patients from Xinjiang. We detected and classified a total of 28 rare variants. DUOX2 mutations were the most frequent DH-associated genetic alterations in Xinjiang Han patients, and we confirmed that these mutations lead to varied genotype-phenotype relationships.

### 4.1. A Limitation of the Current Study

Several limitations should be considered in the interpretation of the present findings. First, the majority of the patients were neonatal and thus were too young to exhibit clinical phenotypes that manifest after three years of age, when L-thyroxine replacement therapy is withdrawn. Therefore, our investigation of genotype-phenotype relationships was incomplete. Second, some patients with heterozygous variants may carry another undetected variant, because NGS-based mutation screening does not detect large noncoding intragenic rearrangements or microdeletions involving one or more exons. Third, in this study, a total of 28 possible pathological variants were identified; all of them were absent in the healthy control. According to the stringent ACMG guideline and based on the available evidence, 10 of them were classified as pathogenic; this will expand the causative mutation spectrum of DH in Chinese patients. However, due to a relatively small sample size and unperformed pedigree analysis in most cases, as well as the lack of functional studies, the evidence is insufficient to support the pathogenicity of the remaining 18 variants; thus, they were classified as likely pathogenic or VUS. This uncertainty would undermine the significance of this study. Therefore, functional studies and further clinical studies with larger cohort sizes will be necessary to elucidate and validate the roles of the mutations identified in this study.

## Figures and Tables

**Table 1:** Clinical phenotypes of DH patients with detected variants.

Patient ID	Age, sex	Birth weight (g)	Gestational age (week + day)	Thyroid widths (cm) (age)	Transient or permanent CH	Screening TSH (*μ*IU/ml)	At confirmative diagnosis, before Tx			Severity	Gene	Variants	
							Age	TSH (*μ*IU/ml)	FT4 (ng/dl)			Nonpolymorphic	Polymorphic
1	2y, M	3550	39 + 6	1.9 (20 d)	NA	15	20 d	80.08	0.84	Mild	DUOX2	p.K530X, p.R1211H	p.H678R, p.S1067L
											DUOXA2	p.R133H	
2^a^	1 y and 10 m, M	3300	37	1.2 (37 d)	NA	33.1	34 d	58.27	0.86	Mild	DUOX2	p.R434_S440del, p.R885Q	
3^b^	3 y and 6 m, M	3400	39 + 6	1 (29 d)	NA	129	29 d	>100	0.38	Moderate	DUOX2	p.S906P, p.L1160del	
4^a^	3 y, F	3900	39 + 1	1.4 (23 d)	NA	122	23 d	>100	0.2	Severe			
5^b^	2 y and 3 m, F	3320	39 + 6	1.4 (27 d)	NA	116	27 d	45.27	0.9	Mild	DUOX2	p.V779M, p.G1513R	
6	4 y and 7 m, F	4440	41	1.1 (8 m)	Permanent	117	35 d	>100	0.32	Moderate	DUOXA2	p.Y138X (Hom), p.Y246X	
7^b^	4 y, F	3400	39 + 4	1.1 (29 d)	NA	318	29 d	>100	0.15	Severe	DUOX2	p.K530X, p.R1110Q	p.H678R, p.S1067L (Hom, CC)
8	5 y and 1 m, F	3100	40	2 (3 6d)	Permanent	56.1	32 d	>100	0.16	Severe	DUOX2	p.K530X, p.R1110Q	p.H678R, p.S1067L (Hom, CC)
9	4 y, F	3365	39	1.3 (16 d)	Permanent	11	17 d	>100	0.3	Severe	DUOX2	p.D435G, p.R1110Q	p.S1067L
10	5 y and 6 m, M	3900	40 + 6	1.16 (52 d)	Transient	39	25 d	39.35	0.61	Moderate	DUOX2	p.K530X, p.R885Q	p.H678R, p.S1067L
11^a^	4 y and 5 m, F	3100	40 + 2	1.08 (47 d)	NA	23.2	33 d	39.73	0.89	Mild	DUOX2	p.M927V (Hom)	
											TG	p.C687LfsX34	
12	1 y and 6 m, F	3800	39 + 1	1.17 (29 d)	NA	69.3	30 d	>100	0.35	Moderate	DUOX2	p.G1513R	
											TG	p.G505D	
13	1 y and 6 m, M	4050	40 + 1	1.1 (33 d)	NA	161	33 d	16.16	0.96	Mild	DUOX2	p.G1521X	
14^a^	4 y and 1 m, F	1600	34 + 2	1 (2 m)	NA	33.6	69 d	37.53	0.93	Mild	DUOX2	p.R885Q, IVS28+1G>T	
15^b^	1 y and 4 m, F	3970	40 + 6	1.5 (26 d)	NA	32.9	27 d	100	0.49	Moderate	DUOX2	p.R432H, p.A1323T, p.L1343F	p.H678R, p.S1067L
16	1 y and 3 m, M	3700	39 + 1	1.3 (23 d)	NA	80	23 d	100	0.14	Severe	DUOX2	p.P76L, p.R683L, p.L1343F	p.H678R, p.S1067L
											TG	p.S1139L	
17	1 y and 2 m, F	3700	40 + 2	2.8 (33 d)	NA	9.12	33 d	>100	0.28	Severe	DUOXA2	p.Y246X	
											SLC26A4	p.A429E	
											TG	IVS10-1G>A	
18^a^	5 y and 11 m, M	4600	40 + 6	1.1 (2 m)	Permanent	100	28 d	100	0.1	Severe	DUOX2	p.R1110Q (Hom)	
19^b^	1 y and 5 m, M	3200	39 + 2	1.4 (42 d)	NA	12.4	37 d	14.5	1.21	Mild	DUOX2	p.R1110Q, IVS28+1G>T	p.S1067L
20	1 y and 3 m, F	2200	34 + 1	1.1 (36 d)	NA	14.9	37 d	14.9	0.51	Moderate	DUOX2	IVS17+1G>T	p.H678R, p.S1067L
											TG	p.G1456R	
21	1 y and 2 m, F	3400	40	1.1 (28 d)	NA	11.7	28 d	64.28	0.55	Moderate	DUOX2		p.H678R, p.S1067L
Normal						0–8		0–8	0.93–1.7				

^a^Neuropsychological development was low; ^b^parental DNA sample available; m: month; d: day; y: year; F: female; M: male; CH: congenital hypothyroidism; TSH: thyroid-stimulating hormone; FT4: free tetraiodothyronine; Tx: L-thyroxine; Hom: homozygous; NA: data not available.

**Table 2:** Potential pathological variants detected in this study.

Gene	Amino acid change	Nucleotide change	Genomic position	Exon/intron position	rs ID	Status^#^	Frequency of variant allele		Reference
							Han Chinese patients (*n* = 21)	Normal control (*n* = 100)	
DUOX2	p.P76L	c.227C>T	chr15:45404850	Exon 4	rs767705906	Known^a^	0.024	0	
DUOX2	p.R432H	c.1295G>A	chr15:45401090	Exon 12	rs530736554	Known^a^	0.02	0	
DUOX2	p.R434_S440 del	c.1300_1320delCGAGATATGGGGCTGCCCAGC	chr15:45401064	Exon 12	NA	Novel	0.024	0	
DUOX2	p.D435G	c.1304A>G	chr15:45401081	Exon 12	rs772040742	Known^a^	0.024	0	
DUOX2	p.K530X	c.1588T>A	chr15:45399648	Exon 14	rs180671269	Known^b^, DM	0.095^∗∗∗^	0	[20]
DUOX2	p.R683L	c.2048G>T	chr15:45398423	Exon 17	rs8028305	Known^b^, DM?	0.024	0	[39]
DUOX2	IVS17+1G>T	c.2148+1G>T	chr15:45398322	Intron 17	NA	Novel	0.024	0	
DUOX2	p.H678R^$^	c.2033A>G	chr15:45398438	Exon 17	rs57659670	Known^b^, DFP	0.19	0.092	[20]
DUOX2	p.V779M	c.2335G>A	chr15:45396563	Exon 19	rs145061993	Known^b^, DM?	0.024	0.005	[11]
DUOX2	p.R885Q	c.2654G>A	chr15:45396158	Exon 20	rs181461079	Known^b^, DM	0.071^∗^	0.005	[20]
DUOX2	p.S906P	c.2716T>C	chr15:45394126	Exon 21	rs768362375	Known^a^	0.024	0	
DUOX2	p.M927V	c.2779A>G	chr15:45394063	Exon 21	rs755186335	Known^a^	0.048^∗^	0	
DUOX2	p.S1067L^$^	c.3200C>T	chr15:45392075	Exon 25	rs269868	Known^b^, DM?	0.286^∗∗∗^	0.085	[20]
DUOX2	p.R1110Q	c.3329G>A	chr15:45391946	Exon 25	rs368488511	Known^b^, DM	0.143^∗∗∗^	0.005	[48]
DUOX2	p.L1160del	c.3478_3480delCTG	chr15:45391615	Exon 26	rs758318135	Known^b^, DM	0.024	0	[11]
DUOX2	p.R1211H	c.3632G>A	chr15:45389873	Exon 28	rs141763307	Known^b^, DM	0.024	0.005	[49]
DUOX2	IVS28+1G>T	c.3693+1G>T	chr15:45389811	Intron 28	rs200717240	Known^a^	0.048^∗^	0	
DUOX2	p.A1323T	c.3967G>A	chr15:45388139	Exon 30	rs550037603	Known^b^, DM	0.024	0	[6]
DUOX2	p.L1343F	c.4027C>T	chr15:45388079	Exon 30	rs147945181	Known^b^, DM?	0.048^∗^	0	[6]
DUOX2	p.G1513R	c.4537G>C	chr15:45386458	Exon 34	rs748262140	Known^a^	0.048^∗^	0	
DUOX2	p.G1521X	c.4561G>T	chr15:45386434	Exon 34	rs765781255	Known^a^	0.024	0	
DUOXA2	p.R133H	c.398G>A	chr15:45408771	Exon 4	NA	Novel	0.024	0	
DUOXA2	p.Y138X	c.413dupA	chr15:45408785	Exon 4	rs778410503	Known^b^, DM	0.048^∗^	0	[43]
DUOXA2	p.Y246X	c.738C>G	chr15:45409472	Exon 5	rs4774518	Known^b^, DM	0.048^∗^	0	[7]
SLC26A4	p.A429E	c.1286C>A	chr7:107334870	Exon 11	rs753269996	Known^b^, DM	0.024	0	[50]
TG	p.G505D	c.1514G>A	chr8:133899131	Exon 9	NA	Novel	0.024	0	
TG	p.C687LfsX34	c.2060_2060delG	chr8:133899676	Exon 9	NA	Novel	0.024	0	
TG	IVS10-1G>A	c.2762-1G>A	chr8:133905934	Intron 10	NA	Known^b^, DM	0.024	0	[51]
TG	p.S1139L	c.3416C>T	chr8:133912567	Exon 15	rs201480815	Known^c^	0.024	0	[22]
TG	p.G1456R	c.4366G>A	chr8:133925498	Exon 20	rs769800036	Known^a^	0.024	0	

^$^Polymorphic variant; ^#^status evaluated based on whether variants are reported in public databases or published literature. ^a^Variants were reported in public population databases, such as dbSNP, ExAC, or 1000 Genomes Project but without phenotypic data and pathological assessment; ^b^variants were reported in the published literature as well as HGMD (professional version 2016.03); DM: disease-causing mutation; DM?: a possible disease-causing mutation; DFP: disease-associated polymorphism with supporting functional evidence; ^c^variants were reported in the published literature; NA: data not available; MAF: minor allele frequency. ^∗^ *P* < 0.05; ^∗∗∗^ *P* < 0.001 according to Fisher's exact test, which compared allelic frequencies of detected variants in patients versus local controls.

**Table 3:** In silico analysis of variants detected in this study.

Gene	Nucleotide change	Amino acid change	SIFT	PolyPhen-2	MutationTaster	FATHMM	M-CAP	MaxEntScan	BDGP	NetGene2
DUOX2	p.P76L	c.227C>T	Damaging	Probably damaging	Disease causing	Tolerated	Possibly pathogenic			
DUOX2	p.R432H	c.1295G>A	Damaging	Probably damaging	Disease causing	Damaging	Possibly pathogenic			
DUOX2	p.R434_S440del	c.1300_1320delCGAGATATGGGGCTGCCCAGC	NA	NA	disease causing	NA	NA			
DUOX2	p.D435G	c.1304A>G	Damaging	Probably damaging	Disease causing	Tolerated	Possibly pathogenic			
DUOX2	p.K530X	c.1588A>T	Damaging	NA	Disease causing	NA	NA			
DUOX2	p.H678R	c.2033A>G	Tolerated	Benign	Polymorphism	Damaging	NA			
DUOX2	p.R683L	c.2048G>T	Damaging	Probably damaging	Disease causing	Damaging	Possibly pathogenic			
DUOX2	IVS17+1G>T	c.2148+1G>T	NA	NA	NA	NA	NA	7.16/−1.35	0.78/−	0.71/−
DUOX2	p.V779M	c.2335G>A	Tolerated	Benign	Disease causing	Damaging	NA			
DUOX2	p.R885Q	c.2654G>A	Damaging	Probably damaging	Disease causing	NA	Possibly pathogenic			
DUOX2	p.S906P	c.2716T>C	Damaging	Probably damaging	Disease causing	Tolerated	Possibly pathogenic			
DUOX2	p.M927V	c.2779A>G	Tolerated	Benign	Disease causing	Tolerated	Possibly pathogenic			
DUOX2	p.S1067L	c.3200C>T	Damaging	Benign	Polymorphism	Damaging	NA			
DUOX2	p.R1110Q	c.3329G>A	Damaging	Probably damaging	Disease causing	Damaging	Possibly pathogenic			
DUOX2	p.L1160del	c.3478_3480delCTG	NA	NA	Disease causing	NA	NA			
DUOX2	p.R1211H	c.3632G>A	Damaging	Probably damaging	Disease causing	Damaging	Possibly pathogenic			
DUOX2	IVS28+1G>T	c.3693+1G>T	NA	NA	NA	NA	NA	8.72/0.22	0.91/−	0.82/−
DUOX2	p.A1323T	c.3967G>A	Damaging	Probably damaging	Disease causing	Tolerated	Likely benign			
DUOX2	p.L1343F	c.4027C>T	Tolerated	Probably damaging	Disease causing	Tolerated	Likely benign			
DUOX2	p.G1513R	c.4537G>C	Damaging	Probably damaging	Disease causing	Damaging	Possibly pathogenic			
DUOX2	p.G1521X	c.4561G>T	NA	NA	Disease causing	NA	NA			
DUOXA2	p.R133H	c.398G>A	Tolerated	Probably damaging	Disease causing	Tolerated	Likely benign			
DUOXA2	p.Y138fs	c.413dupA	NA	NA	Disease causing	NA	NA			
DUOXA2	p.Y246X	c.738C>G	NA	NA	Disease causing	NA	NA			
SLC26A4	p.A429E	c.1286C>A	Damaging	Probably damaging	Polymorphism	Damaging	Possibly pathogenic			
TG	p.G505D	c.1514G>A	Damaging	Probably damaging	Disease causing	Tolerated	Likely benign			
TG	p.C687LfsX34	c.2060_2060delG	NA	NA	Disease causing	NA	NA			
TG	IVS10-1G>A	c.2762-1G>A	NA	NA	NA	NA	NA	NA	0.99/−	0.94/0.17
TG	p.S1139L	c.3416C>T	Damaging	Benign	Polymorphism	Tolerated	Likely benign			
TG	p.G1456R	c.4366G>A	Tolerated	Benign	Polymorphism	Damaging	Likely benign			

SIFT, PolyPhen-2, MutationTaster, FATHMM, and M-CAP were used to predict the effects of missense and indel mutations; MaxEntScan, BDGP, and NetGene2 were used to predict the damaging effects of splicing mutations with a wild-type/mutant score; − means depletion of the 5′ splice site; NA: not available.

## Data Availability

The data used to support the findings of this study are available from the corresponding author upon request.

## Supplementary Materials

Supplementary 1 Supplementary Figure S1: evolutionary conservation analysis of detected novel mutations (missense and indel) based on multiple sequence alignment from different species (human: *Homo sapiens*; GORGO: *Gorilla gorilla gorilla*; PAPAN: *Papio anubis*; MACMU: *Macaca mulatta*; bovine: *Bos taurus*; pig: *Sus scrofa*; CANLF: *Canis lupus familiaris*; rat: *Rattus norvegicus*; mouse: *Mus musculus*). (A) DUOX2: p.R434_S440del (highly conserved); (B) TG: p.G505D (highly conserved); (C) DUOXA2: R133H (highly conserved).

Supplementary 2 Supplementary Table S1: customized primers and amplicons for seven targeted genes. Fwd: forward; Rev: reverse; ^a^physical position of the amplicons was obtained from the human assembly (GRCh37/hg19). Supplementary Table S2: PCR primers used to validate gene sequence variants. Supplementary Table S3: Ion Torrent PGMTM statistics and potential disease variants in patients with CH. Q20: 99% certainty that the correct base was called. Supplementary Table S4: classification and evidence of 30 variants. P: pathogenic; LP: likely pathogenic; VUS: variants of uncertain significance; B: benign; D: damaged; T: tolerated; NA: not available; CHB: Han Chinese in Beijing, China; PVS1: null variant (nonsense, frameshift, canonical ±1 or 2 splice sites, initiation codon, and single or multiexon deletion) in a gene where LOF is a known mechanism of disease; PS3: well-established in vitro or in vivo functional studies supportive of a damaging effect on the gene or gene product; PS4: prevalence of the variant in affected individuals is increased compared with controls; PM1: located in a mutational hot spot and/or critical and well-established functional domain; PM2: for recessive disorders, extremely low frequency in 1000 Genomes Project or Exome Aggregation Consortium (ExAC); PM3: for recessive disorders, detected in *trans* with a pathogenic variant; PM4: protein length changes as a result of in-frame deletions/insertions in a nonrepeat region or stop-loss variants; PP3: multiple lines of computational evidence support a deleterious effect on the gene or gene product (detailed prediction results shown in Table 3 and Table S4); BA1: allele frequency is >5% in 1000 Genomes Project, Exome Aggregation Consortium (ExAC), or control population. Supplementary Table S5: clinical characteristics of DH patients in the validation cohort (*n* = 32) and the validated variants. m: month; d: day; y: year; F: female; M: male; CH: congenital hypothyroidism; TSH: thyroid-stimulating hormone; FT4: free tetraiodothyronine; Tx: L-thyroxine; Hom: homozygous; NA: data not available.
