# Supplementary material for: Mutational Spectrum Analysis of Seven Genes Associated with Thyroid Dyshormonogenesis
Source: Int J Endocrinol. 2018 Aug 2;2018:8986475. doi: 10.1155/2018/8986475 (PMC6098846; doi:10.1155/2018/8986475)
Supplement: Supplementary 2 — Supplementary Table S1: customized primers and amplicons for seven targeted genes. Fwd: forward; Rev: reverse; aphysical position of the amplicons was obtained from the human assembly (GRCh37/hg19). Supplementary Table S2: PCR primers used to validate gene sequence variants. Supplementary Table S3: Ion Torrent PGMTM statistics and potential disease variants in patients with CH. Q20: 99% certainty that the correct base was called. Supplementary Table S4: classification and evidence of 30 variants. P: pathogenic; LP: likely pathogenic; VUS: variants of uncertain significance; B: benign; D: damaged; T: tolerated; NA: not available; CHB: Han Chinese in Beijing, China; PVS1: null variant (nonsense, frameshift, canonical ±1 or 2 splice sites, initiation codon, and single or multiexon deletion) in a gene where LOF is a known mechanism of disease; PS3: well-established in vitro or in vivo functional studies supportive of a damaging effect on the gene or gene product; PS4: prevalence of the variant in affected individuals is increased compared with controls; PM1: located in a mutational hot spot and/or critical and well-established functional domain; PM2: for recessive disorders, extremely low frequency in 1000 Genomes Project or Exome Aggregation Consortium (ExAC); PM3: for recessive disorders, detected in trans with a pathogenic variant; PM4: protein length changes as a result of in-frame deletions/insertions in a nonrepeat region or stop-loss variants; PP3: multiple lines of computational evidence support a deleterious effect on the gene or gene product (detailed prediction results shown in Table 3 and Table S4); BA1: allele frequency is >5% in 1000 Genomes Project, Exome Aggregation Consortium (ExAC), or control population. Supplementary Table S5: clinical characteristics of DH patients in the validation cohort (n = 32) and the validated variants. m: month; d: day; y: year; F: female; M: male; CH: congenital hypothyroidism; TSH: thyroid-stimulating hormone; FT4: free tet [file 8986475.f2.doc]

Supplementary Table S1: Customized primers and amplicons for seven targeted genes.

| Amplicon_ID | Ion_AmpliSeq_Fwd_Primer* | Ion_AmpliSeq_Rev_Primer* | Gene Name | | Location^a^ | Length(bp) | Accession Number |
| --- | --- | --- | --- | --- | --- | --- | --- |
| AMPL7155234972 | CTGAGGCCTAAGGTGGATTCTG | CTGGGCTTGAAGCCTTAGTCATT | DUOX2 | | chr15:45386240-45386579 | 339 | NM_014080.4 |
| AMPL7155234979 | CCTTTAACAGCTGACCTCATCCT | CCCATTCTCTGTCTCTTGGGCTA | DUOX2 | | chr15:45386621-45386979 | 358 |  |
| AMPL7155234984 | GCAGGTCAGACCAAAGACAGTC | ATCCAAGAGGTGGAGGAGAACGA | DUOX2 | | chr15:45386904-45387232 | 328 |  |
| AMPL7153134585 | GGGTGACATAAATGTGCACAGACA | GGATCTAGGGAGGCTGAACTGA | DUOX2 | | chr15:45387172-45387344 | 172 |  |
| AMPL7155235005 | GGCATCTCAACCACTGGGTAAG | TGTCTCCGTTTCTGGTCTCAGA | DUOX2 | | chr15:45387494-45387861 | 367 |  |
| AMPL7155234995 | GATAGGGAAGGGCAGAGATCCT | GGTGCCTATCAGTCCTCAGGTA | DUOX2 | | chr15:45387958-45388329 | 371 |  |
| AMPL7155435722 | CCACCTCTTCTCAGGTCACTTC | CCCACTTTCCACATCTACTTCCTG | DUOX2 | | chr15:45389195-45389553 | 358 |  |
| AMPL7155415877 | CCAGCTTGTCACCTCCATAGATG | CCAAGATCTCAGAATGAGACTTTGAGATT | DUOX2 | | chr15:45389496-45389733 | 237 |  |
| AMPL7155235006 | GGGTCTTAAATCTCAAAGTCTCATTCTGA | ATGAGAGAGGAAGGAGGGAAGGATAG | DUOX2 | | chr15:45389697-45390040 | 343 |  |
| AMPL7155234983 | GGCTTGTCCTGAAGGCTAGAAA | TACAGAGGGTGGAACCATGGAA | DUOX2 | | chr15:45390087-45390437 | 350 |  |
| AMPL7155235010 | CGGCCTGCCTATTTCTTTTTCTATT | CCTTATACCTCCATCTCTCCTCCCTAT | DUOX2 | | chr15:45391503-45391781 | 278 |  |
| AMPL7155435721 | GAGTGGGCTGACTGAGAAGATG | CATGTTCTCTTATATCTTGCTCACCATGT | DUOX2 | | chr15:45391625-45391999 | 374 |  |
| AMPL7155415875 | GAAAGTCTCTCGCAGGAAGGTTAT | CCCTCTGATCCTAAGAGTCTGCAT | DUOX2 | | chr15:45391936-45392181 | 245 |  |
| AMPL7155235002 | CTCTCCCATGCAGACTCTTAGG | GTCACCTTTCATGGAGTGGCAT | DUOX2 | | chr15:45392151-45392504 | 353 |  |
| AMPL7155234988 | GGAACAAATGGAATGAGACTCAGGAT | GCCAGAATCATTCTACAAAACAAATCATGA | DUOX2 | | chr15:45392832-45393205 | 373 |  |
| AMPL7155235001 | GCAGCTGGAATGTTTGTGCTAC | AGTGAGTGCTTAGTCTAGGGAATGA | DUOX2 | | chr15:45393257-45393621 | 364 |  |
| AMPL7155234980 | CCTCTTCCTCTGGCCAGATACAT | GCTGACATGGGAGGATTTTCACTT | DUOX2 | | chr15:45393719-45394088 | 369 |  |
| AMPL7155234981 | CACCTCCACCTTTGACACAGAG | GTCACTGGTAACCACATTAGATAATCTGG | DUOX2 | | chr15:45394003-45394292 | 289 |  |
| AMPL7155235017 | CCAGTCAGGTAAATATTTGAAGGTGGA | CCTGGTGGTCTTCATGAAAGGTAG | DUOX2 | | chr15:45395984-45396357 | 373 |  |
| AMPL7155234996 | GCAGGTCATCTCCTTGCTGAAA | ATATGAAGGGTCACTGAACTGCTTC | DUOX2 | | chr15:45396272-45396643 | 371 |  |
| AMPL7156642760 | CCATAGAGCGGAAGCTTAGTTCA | CCCTCAACCCAAGATCCATTGA | DUOX2 | | chr15:45397737-45398111 | 374 |  |
| AMPL7155235004 | GGCCTCGCTTGTGATAATGGAG | GACTCACAGAGGAATTGACCTGAAG | DUOX2 | | chr15:45398257-45398604 | 347 |  |
| AMPL7155234977 | CGCCTTCAGGTCAATTCCTCTG | GTTTGAGACCAGGCTCAAGGAA | DUOX2 | | chr15:45398577-45398949 | 372 |  |
| AMPL7155234982 | GTTCCTTGAGCCTGGTCTCAAA | GAAGACAGATGATACCTTGGTCCTT | DUOX2 | | chr15:45398927-45399270 | 343 |  |
| AMPL7155435713 | CCTCCTAGCCCAACACAGAAG | CCCTCTGGGTCTCTTTTCTCAC | DUOX2 | | chr15:45399421-45399760 | 339 |  |
| AMPL7153212232 | GGGCTGTCTAAGGCTAGACA | CCAGGACCTATCCCAGCTAGA | DUOX2 | | chr15:45400167-45400391 | 224 |  |
| AMPL7153212237 | CGTACAAACTGGTCGAGGACAA | GCTTGCCTAAGAAGAGGAAATCAGG | DUOX2 | | chr15:45400290-45400556 | 266 |  |
| AMPL7155235016 | AATGAGTCAACATATGTAAAGGGTTTGGA | GTGCCAATAGTCAGATACAAGGCT | DUOX2 | | chr15:45400903-45401233 | 330 |  |
| AMPL7155234975 | ATTTTCTTCTTATGGCTCCTTGAAGTCT | GGGACAACAGTTCAAGAGAGTCT | DUOX2 | | chr15:45401578-45401951 | 373 |  |
| AMPL7155234987 | CCCGGATAATTTCCTCTACCCTTCA | AATCTCTGCCGTGTTCAAGGAA | DUOX2 | | chr15:45401950-45402315 | 365 |  |
| AMPL7155235007 | GAATGACCCAGCTGCTAGACAA | CAGGATACCGTCCTTTCCTAGAC | DUOX2 | | chr15:45402384-45402725 | 341 |  |
| AMPL7155235008 | CTGCTCAGAGGCCACCACAAAT | TGACCCTTCCTGGTCTCATCTC | DUOX2 | | chr15:45402664-45403016 | 352 |  |
| AMPL7155235019 | GGTGATAAATAATCATCACCAGCCAGAT | GTTCCGCTACCACAACCTGT | DUOX2 | | chr15:45403103-45403417 | 314 |  |
| AMPL7153212168 | TAGGTGGCGATGACCCTCT | GATCACGCTACCGCTCGT | DUOX2 | | chr15:45403313-45403524 | 211 |  |
| AMPL7155234991 | GAGTGCGAGGAGCCATAGAT | CCGAGTTCCTCAACATCCGC | DUOX2 | | chr15:45403728-45404102 | 374 |  |
| AMPL7155399284 | CAGGTGGGATGCGGATGTT | CGCAATACGGACGGTTTGTC | DUOX2 | | chr15:45404073-45404198 | 125 |  |
| AMPL7155234973 | CTGAGCAGAGCGCCAGATCAAC | GGTTCTGTCAGTGAAGTGGGAT | DUOX2 | | chr15:45404703-45405023 | 320 |  |
| AMPL7155235009 | GGCTCTGTCTAAGCACTCCAT | AGTCAGGACGCACTCTCACT | DUOX2 | | chr15:45404936-45405272 | 336 |  |
| AMPL7153212528 | AGGTTGTTAAACCAGCCGTCATAG | CCTGAGACTGGAAGGTCATTTCG | DUOX2 | | chr15:45405211-45405457 | 246 |  |
| AMPL7155235003 | GCGAAATGACCTTCCAGTCTCA | TCCAGTACTAGCTGTAGGAACAAGAG | DUOX2 | | chr15:45405434-45405718 | 284 |  |
| AMPL7155223002 | CCAGGAAAGTAACGGCTACAGA | GCTTGCTGCTAGAGCCAAAAAC | DUOXA2 | | chr15:45406541-45406914 | 373 | NM_207581.3 |
| AMPL7156640855 | GCGTTCCACTGCTCATCGTTAT | AGCCTCAATTCGACTATTCATGGAG | DUOXA2 | | chr15:45406865-45407069 | 204 |  |
| AMPL7155221462 | CCAGAGGGACTTGCAGGAAAAG | CATGGTAAGGGCCAAAAAGTGG | DUOXA2 | | chr15:45407850-45408206 | 356 |  |
| AMPL7155221429 | CATGCAACCACATTGGACCTCTA | ACGAGGACCACAAGCTATTTGAG | DUOXA2 | | chr15:45408203-45408576 | 373 |  |
| AMPL7155221453 | GGGCCTGGAGGGCATTAATATT | CCAGGTGAACTGCTCGTTGTAG | DUOXA2 | | chr15:45408425-45408769 | 344 |  |
| AMPL7155221454 | GCATCAGCTGAACGAGACCATT | GCAGATCTCGTGGATCTTCTAACC | DUOXA2 | | chr15:45408724-45409091 | 367 |  |
| AMPL7155221433 | GGATAAAGAAGAGCGCAGCTGT | CTGTAAACTGGAAAGTGCACACC | DUOXA2 | | chr15:45409208-45409582 | 374 |  |
| AMPL7155223000 | GGGTAGGCTCCAAAAGATGGAA | CCCTCACAGGTTAGTGGTGATAC | DUOXA2 | | chr15:45409748-45410110 | 362 |  |
| AMPL7154275000 | GCCGCTCTCCCAGACTTAAAAT | AGCCATGAAAATTAATGGGAAAAACAGTTT | DUOXA2 | | chr15:45410066-45410331 | 265 |  |
| AMPL7155234963 | CGGACATCGACAGCCCATA | ATGCCAGTGGACACCAGGA | SLC5A5 | | chr19:17982930-17983217 | 287 | NM_000453.2 |
| AMPL7155232627 | CCTTCGGAGCCTGGGACTA | TGAGGACCGAGTTCAGAAGCT | SLC5A5 | | chr19:17983160-17983429 | 269 |  |
| AMPL7155232628 | TATGGCCTCAAGTTCCTCTGGAT | CATCCGCGTCCTCCTGTA | SLC5A5 | | chr19:17983375-17983591 | 216 |  |
| AMPL7155234970 | GTGCACACGCATGGACCAGT | CGGGCTAGTGAAGGCTGA | SLC5A5 | | chr19:17984723-17985074 | 351 |  |
| AMPL7153508092 | CCATATTCTGGGACCACCCTTCT | CGGTCACTATGCAAAAAGAGGTC | SLC5A5 | | chr19:17985211-17985478 | 267 |  |
| AMPL7155234967 | CTCATCCTGAACCAAGGTGTGA | CAGCAATAAGCTTTTCAGATCATTTTTCAC | SLC5A5 | | chr19:17985339-17985703 | 364 |  |
| AMPL7155433065 | AACTTAATTCTTACAACAACACAGTAAGAAGG | CCCATCCTGCTGGAGTATCC | SLC5A5 | | chr19:17986603-17986952 | 349 |  |
| AMPL7156640661 | CCAACAAAACCCACTCCAAATGT | CATACATGGAGAGCCACACCAA | SLC5A5 | | chr19:17988415-17988611 | 196 |  |
| AMPL7155433062 | CGCTATACATTCTGGACTTTTGTGG | CAGTGTAGAACACAAACATGACGATG | SLC5A5 | | chr19:17988554-17988858 | 304 |  |
| AMPL7155234959 | ACAAAACCACAGACAAAAACTAAATGCA | TCTTCTAGGGATCAAGCAGGAACT | SLC5A5 | | chr19:17991523-17991897 | 374 |  |
| AMPL7155234960 | GGGCAAATATCTCCTTCACCTTTG | CGATCCGTAGATGAGTGCTGTA | SLC5A5 | | chr19:17992631-17992974 | 343 |  |
| AMPL7155234964 | GCATCCACCAGCATCAATGCTA | GAAGTGAGTTCTGCCACTGACA | SLC5A5 | | chr19:17992773-17993143 | 370 |  |
| AMPL7155234961 | GAGTTCCTGAGGTCTCGCTT | CAGGACCCTCATGGTCTGC | SLC5A5 | | chr19:17994396-17994757 | 361 |  |
| AMPL7155234971 | GGGAGCCTTCATCTTGGGAAT | CCCTACCCTCAAGTCTGCTCA | SLC5A5 | | chr19:17994531-17994877 | 346 |  |
| AMPL7155231906 | GCGTGGCTCTCTCAGTCAAC | CCCTTCAAGATCACACCATCCA | SLC5A5 | | chr19:17994777-17994913 | 136 |  |
| AMPL7155234957 | GGATACCTGGAGGGAAGGAACAT | GCCTTGAGGCAGAGAGATGAAT | SLC5A5 | | chr19:17998965-17999323 | 358 |  |
| AMPL7155234968 | GGGACTGGAGCCTGTTAGACTT | AATAGAGTCTTTTGACTGGAGATAATGAGC | SLC5A5 | | chr19:18001571-18001943 | 372 |  |
| AMPL7155234956 | CCAGGTCATCAGTAGCCCATCT | TACAAGGCAATCCAGCCAATCA | SLC5A5 | | chr19:18004427-18004794 | 367 |  |
| AMPL7155234732 | GCAGTTTCTGTAACCTCCTGACA | AGGAGCTACCATTATGCCCTCTA | TPO | | chr2:1417986-1418359 | 373 | NM_000547.5 |
| AMPL7153464249 | GCAAGATGGGCTTGAGGAACAA | TCATCTGCAATTGCGAAAATCAGG | TPO | | chr2:1426729-1427000 | 271 |  |
| AMPL7155234726 | GTCACATTGTCTGGGACACAGTA | CACAAAGTCAAGGTGTCCTCCTT | TPO | | chr2:1437092-1437444 | 352 |  |
| AMPL7155234742 | CCCAGTTACATATGAATCCCAAATTCAG | ATACAGATCCACCAGTGGTGGTA | TPO | | chr2:1439904-1440257 | 353 |  |
| AMPL7155234750 | CCACTTATTCTCCCTGAGAATGGT | CTGGGAGGATCTGAAGGGATTG | TPO | | chr2:1457367-1457722 | 355 |  |
| AMPL7155234745 | CCAGGAAGTGCATGATCCCAAA | CCATTCTGTTTGACGTTTTAAATAGCACT | TPO | | chr2:1459763-1460123 | 360 |  |
| AMPL7155234737 | GCCTCGAACTTCCAGAGTCTTA | CGAGGTCAACCCGTTCATCT | TPO | | chr2:1480785-1481004 | 219 |  |
| AMPL7155234738 | GAACCTGTCCACGGCCAA | CTCCAGTGCGCATTGAGG | TPO | | chr2:1480956-1481324 | 368 |  |
| AMPL7154067858 | CTCAAGGCCCTCAATGCG | CTGGGAGAGAGAAGCCACGATG | TPO | | chr2:1481299-1481469 | 170 |  |
| AMPL7155234727 | TGGGAAGGAGGCTCTCTAGTC | CTGTGGAGAACACGTTGGACA | TPO | | chr2:1488143-1488491 | 348 |  |
| AMPL7155234728 | TCCCTATGAAGGCTATGACTCCA | GCACATGTATACCCTACGTAACAAACC | TPO | | chr2:1488433-1488758 | 325 |  |
| AMPL7155234751 | ATTGTTGTTTCTCTAGAACTGAGCCAA | TCTCTCTAGCAGCAGGTTGCTA | TPO | | chr2:1491532-1491841 | 309 |  |
| AMPL7155234731 | GGACCATGGCATGAGTGAGATG | TCTGGATAGGAACGTACCAGTCA | TPO | | chr2:1497496-1497828 | 332 |  |
| AMPL7155231694 | TGTCTCATTGGGAAGCAGATGAAG | GCTTACGTAAGAGTTGAATGGTGGA | TPO | | chr2:1497768-1497934 | 166 |  |
| AMPL7155433050 | CATCTCCTTCGGGTCCTCAGTA | CGTGAAGACGTGGCTGTTCT | TPO | | chr2:1499447-1499791 | 344 |  |
| AMPL7157651842 | CTTACTCACTGTCTCCTTCTCTGGA | TGATGAGATGCACGTGCTGTAA | TPO | | chr2:1499735-1500019 | 284 |  |
| AMPL7156642706 | CGTTGGTGTGTGGTTTTCTTTTCTC | GCGAAGAAATTTTCAATGACATTGCATT | TPO | | chr2:1500304-1500624 | 320 |  |
| AMPL7155234730 | GCTCCTCATCACCTTTTCGGAT | TCTTCATTTTACAAAAACTCGCAAATGGA | TPO | | chr2:1507537-1507909 | 372 |  |
| AMPL7155234747 | CTTTTCTGGGCAGATAAATGTCCCT | TTTGAGATACCAGTAACCTGTGTTAGC | TPO | | chr2:1520499-1520873 | 374 |  |
| AMPL7155234741 | GGAGTGTTCCTGCCAAAGACAA | GGACTAGGAAACACGGAGTAAACAG | TPO | | chr2:1544248-1544616 | 368 |  |
| AMPL7155234746 | CCTCCAGCATGACAAGCAAGAA | CATTTATTCATCCATGCCTGAGAGTAACTA | TPO | | chr2:1546037-1546410 | 373 |  |
| AMPL7155234945 | ACACCTGTGTCTGCATTCCTTC | CCATAAGCCCTGCCATTGACTC | IYD | | chr6:150690059-150690425 | 366 | NM_203395.2 |
| AMPL7155232622 | TGCACCCAGCTGGAGTTTTAAG | CCTTCTCAGGATAGTGGTTATGAGAGAAG | IYD | | chr6:150710295-150710562 | 267 |  |
| AMPL7155413470 | GATGAATGGCAAGAATCAGAAGAAAATGTT | ACACCAGTAAGGTGACTAACATTTCC | IYD | | chr6:150710493-150710730 | 237 |  |
| AMPL7155234938 | CTACAGGGATGAGCCTCTCCTTA | TTACAACAAAGCCAGGACATGGT | IYD | | chr6:150713398-150713726 | 328 |  |
| AMPL7155234952 | GACAAAAACAAAAATTCTGCAGCTTTTCA | TGTCCCTGAATTGAAAACTCAGCT | IYD | | chr6:150715089-150715463 | 374 |  |
| AMPL7155234942 | GCTTCTTCACTATCTCAAAGAAAAACATGT | GCTTCATGGAGATTTTTCTTTCCTCCA | IYD | | chr6:150716453-150716827 | 374 |  |
| AMPL7155234941 | AGGTTAGAGGGAGAGCAGTCAT | GACCTGACACCTGGAGAAAGAG | IYD | | chr6:150719125-150719481 | 356 |  |
| AMPL7154850868 | GGGCTTACTCGCTTCAAGTT | GAAAGCGAGCTCGCTGTAGA | SLC26A4 | | chr7:107301812-107302182 | 370 | NM_000441.1 |
| AMPL7154850869 | CGAGTACAGCTGCAGCTACATG | AGTTTCCCAGGTAAGTTCATTTCGG | SLC26A4 | | chr7:107302128-107302478 | 350 |  |
| AMPL7154850864 | AAACATCAGCAGAATCCAGTTCATAAC | GGTAAGCAACCATCTGTCACAGA | SLC26A4 | | chr7:107303647-107303973 | 326 |  |
| AMPL7154850837 | CATATTGCTTTTGCATCATCATAAAGGC | TTGCACCAACCTAATAGAGGTATAATGC | SLC26A4 | | chr7:107312465-107312785 | 320 |  |
| AMPL7154850861 | AACCCTATGCAGACACATTGAACA | GGGTTCCAGGAAATTACTTTGTTTTGTTTT | SLC26A4 | | chr7:107314539-107314908 | 369 |  |
| AMPL7154850842 | GTATTTTTGTGCTATAGGCAGGCTACTA | GGCCCAGACTCAGAGAATGAAT | SLC26A4 | | chr7:107315265-107315632 | 367 |  |
| AMPL7154850830 | GTGGGAAGATTCATATGAGAATTGATTGTG | GCAGTAGCAATTATCGTCTGAAATAAAACA | SLC26A4 | | chr7:107323546-107323916 | 370 |  |
| AMPL7154850840 | ATGATCGGTTTAGACACAAAATCCCA | AGATCACACACAAATAGGACTATTGAAGG | SLC26A4 | | chr7:107323747-107324112 | 365 |  |
| AMPL7154850857 | CTTTCATGTCTAATATGTGACTGAGCAGA | CAATAAGGTCTGGTGAAAGAATCCAAC | SLC26A4 | | chr7:107329394-107329764 | 370 |  |
| AMPL7154850833 | GCAGAGTAGGCATGGGAGTTTT | TTAGGAAGTACAAGTCTGAAAGGCAAATT | SLC26A4 | | chr7:107330460-107330798 | 338 |  |
| AMPL7154850838 | TGAGCAAATAACTATCACTTTTCTCGACA | AGAGAGACAGAGAAGGCTGTGTTA | SLC26A4 | | chr7:107334685-107335059 | 374 |  |
| AMPL7154850828 | GGTATAACCCTGCTTCTCTGCA | CCACAATTCTAATTTCTCCTCTGGAGTT | SLC26A4 | | chr7:107334925-107335297 | 372 |  |
| AMPL7152997946 | AGGTAGTTATCACATGATGGTACCTGA | CAAAATGGTATAAGGAAGCTCAGAGTGT | SLC26A4 | | chr7:107336310-107336553 | 243 |  |
| AMPL7154850846 | TTCAGAGTTAGCTACAGGAAAATGTCATC | CACTGGATCAAAAGTTTTCATGACACTC | SLC26A4 | | chr7:107338336-107338710 | 374 |  |
| AMPL7154850867 | GAAAAGAAAGAAAAGTTGAGTGCTGCTA | CTCATTGCCCTACACAAAGGGA | SLC26A4 | | chr7:107340389-107340747 | 358 |  |
| AMPL7154850820 | AGATCTACTCCATCAGACCTTACAATTTCT | TGATACGTACATGTCATTTGTTCATTCACA | SLC26A4 | | chr7:107341396-107341770 | 374 |  |
| AMPL7157604914 | ACTACCCACCATAGAAGGCAGT | ATCAGGCTCAAAAGCATTATTTGTTGAA | SLC26A4 | | chr7:107341951-107342322 | 371 |  |
| AMPL7157775959 | GTGTTTTCTTCGTTTAGAATGGCATCATA | CATTTTACTATTGCCAAAGCTCCAAATGT | SLC26A4 | | chr7:107342255-107342556 | 301 |  |
| AMPL7154850825 | TTTGAAAACCTCCATGGTTTTGCA | GGAATGAAGCAGTGCCAGATTC | SLC26A4 | | chr7:107344619-107344992 | 373 |  |
| AMPL7154894585 | TGGAAAAGAAACTGTCATTTCAAATCTGG | CATTGCTCCAGCTTTTCTATCACATAAT | SLC26A4 | | chr7:107350175-107350526 | 351 |  |
| AMPL7152997850 | TTTCCTAGGAACTAACAAAACATTGTGTCT | AGACTAGACTTGTGTAATGTTTGCCATTTA | SLC26A4 | | chr7:107350458-107350728 | 270 |  |
| AMPL7154850823 | AGAACAATACAGCTGAAGAGGATTCTG | GCTATGAAGCCATCTTTTCTGCATTTG | SLC26A4 | | chr7:107352848-107353203 | 355 |  |
| AMPL7154850848 | ACAGTGAGTGAGATTCAGTCTCCA | GCCTAGAAGCAGTCTTAGTGCT | SLC26A4 | | chr7:107355660-107356031 | 371 |  |
| AMPL7155234780 | GCTCCTTTTGACCAGCAGAGAA | AGCAAATCACTGACATGTGGGAA | TG | | chr8:133879085-133879441 | 356 | NM_003235.4 |
| AMPL7156642724 | AAAGTGTGCTTAGCTGCCAAATTT | GCCTCACATGCACAAAGGAGTT | TG | | chr8:133880213-133880581 | 368 |  |
| AMPL7155234842 | CTCCACTCTCTCCCTAATTTAAACGAAC | GGGATGATTCTTATTCACAGAGGAGAAG | TG | | chr8:133881839-133882213 | 374 |  |
| AMPL7155234783 | GGGAAGGGAGCATGAGTTTTCC | CAGGAAACCCTAAATGTGTTGGATC | TG | | chr8:133883509-133883883 | 374 |  |
| AMPL7155234825 | GCTAAGGGACACGAGTGCATAT | AGCTCTTAATTCCTTATCATGCTGCA | TG | | chr8:133885219-133885581 | 362 |  |
| AMPL7155234788 | CCTTTTCACTAGGCGTGGACTT | GCTAAGGTTGTGTCCTCCTCAATG | TG | | chr8:133894007-133894363 | 356 |  |
| AMPL7155234809 | GGTGAAGGCTGTCTCTCTCAGT | TCTTTTCATCTCTGCCCTCACTTTG | TG | | chr8:133894617-133894973 | 356 |  |
| AMPL7155234841 | GTTAAGAGCAAACAGCAGTGCAT | ACACTAGAAGGCAGTTGAATTAATGGG | | TG | chr8:133894977-133895350 | 373 |  |
| AMPL7155433014 | TGGTTTCAAACGTAGGTGTCCTT | CCACAAAGAGCTCCTTGATCGT | | TG | chr8:133898566-133898869 | 303 |  |
| AMPL7155433015 | AAGAGTAGCCAGATTTGCCACAT | GTTAAATGTGCCTCTTGTGCCAA | | TG | chr8:133898814-133899105 | 291 |  |
| AMPL7155433016 | GGTGAATGTTGGCCAGTTTAACTT | CCGTTTCCATCACATCACCTAAATCT | | TG | chr8:133899048-133899421 | 373 |  |
| AMPL7155433017 | CAACATGCTATCTCTGTGCCAGAA | GTAGCACTCTGAGTTGAAGCACT | | TG | chr8:133899361-133899729 | 368 |  |
| AMPL7155413419 | CTGCTTGTACTAGTGAGGGACATTT | GATTTCTATTACTGTACTGCATTGGGTCA | | TG | chr8:133899671-133899875 | 204 |  |
| AMPL7155234771 | TGATGTCAATGGTGCTGATTTAGTCA | CGTTGGTACAACTCGAAGACCT | | TG | chr8:133900038-133900412 | 374 |  |
| AMPL7155234772 | GGAGACAAGTGCAATGCAATGG | GCTCCAGGAGGATATTCTCCCT | | TG | chr8:133900358-133900645 | 287 |  |
| AMPL7155234773 | CTAGCAGCACTGGAAGGGAAAC | TACCCTCCCACTAGACATCCTTTTT | | TG | chr8:133900591-133900910 | 319 |  |
| AMPL7155234834 | TTCATGGGAGTCAGAGGGAAGT | GAGAGACGAATTTAAGCTCCTGCTT | | TG | chr8:133905869-133906243 | 374 |  |
| AMPL7155234850 | TAAATAAGAAGGCCTCCTTATGTTGGAAC | GCTAAAGAGGAGCCTAAGAGTCCA | | TG | chr8:133909782-133910143 | 361 |  |
| AMPL7155234781 | GGCTTGCACTGCTATGAGTGAA | CCTTCCAACTAGGAAGTGCCAA | | TG | chr8:133910312-133910684 | 372 |  |
| AMPL7155234784 | TGTCCTCAGAGATTCTACCTCTCTATGA | CCCAGGCTGAGAACATCATGTG | | TG | chr8:133910901-133911275 | 374 |  |
| AMPL7155234849 | CCACATCTCTCCGTCTGGAGAA | CCCACAGCTATGGAAGCAGTAG | | TG | chr8:133912352-133912695 | 343 |  |
| AMPL7155234843 | TCTCTCTTCCTCTGTAGGCTCCTA | GGACTCCACTCTTGAGCACATTG | | TG | chr8:133913272-133913633 | 361 |  |
| AMPL7155234844 | CATCTGGCTTGTCTCTGTGTCA | AGTCTGGATCAAAGAACAGATATGCAG | | TG | chr8:133913575-133913858 | 283 |  |
| AMPL7155433012 | GCAGATGGGCTCATCCAAGTAG | TATCAATGGCCCTGGTGGAAAC | | TG | chr8:133918731-133919087 | 356 |  |
| AMPL7155433013 | GGAGGTGGTTGGTGGAACAATC | GAAAATGCTCCATGGGACTCTCT | | TG | chr8:133918964-133919310 | 346 |  |
| AMPL7155234776 | TTTTGAGCTCAATGAGGAAGGGTT | CTGTCTCCAGCCTCTGTGTTAAT | | TG | chr8:133920326-133920685 | 359 |  |
| AMPL7155234854 | TGGAGGATTGAAGGAAGTAACATAAGC | ACAAGAAGACATCATGGGCTTCTTC | | TG | chr8:133923512-133923886 | 374 |  |
| AMPL7155234852 | GGCATCTCTGCCCTGCTTAATC | GCTGAGGTGGCCTTTCCTAAAG | | TG | chr8:133925211-133925578 | 367 |  |
| AMPL7155234847 | TGAATGAGCTCTTGAACTGGTTTGA | CTGCACAAAGAATTATCTGTCCCAAAAT | | TG | chr8:133931510-133931884 | 374 |  |
| AMPL7155234792 | GTCTGAGTTAGATGTGTGACTTGGA | CCTCCAGCTTCTCTCCCTTTCTAA | | TG | chr8:133935469-133935840 | 371 |  |
| AMPL7155234806 | GTATGTTGGAGCTACCTTACAAAGGA | GTTTTAATGGTTTGATTTGGTTTGTCTCGA | | TG | chr8:133941158-133941531 | 373 |  |
| AMPL7155234833 | ATTTGTGTATCTGCATATTTGTGTGTGTG | ATCAGCTCTCGACAACCTCATTC | | TG | chr8:133945661-133946035 | 374 |  |
| AMPL7156642725 | GGCTGCAAGTTACTGCTTACAC | GCTGGAAACCAGCTCCAAACAT | | TG | chr8:133947837-133948181 | 344 |  |
| AMPL7155234769 | CTGTCCAACTCTGCCATGTTTTG | GTGTAGGCACTCACACTGTACTC | | TG | chr8:133953538-133953901 | 363 |  |
| AMPL7155234824 | CCTGAGACGCTGTCACCTATCT | CCACAAGGAGGGCTCATAAGAA | | TG | chr8:133960910-133961278 | 368 |  |
| AMPL7155234818 | GCTGCAAGAAGCTTCTGGGTTA | TGGAGCCCTTCTATTCCACAGA | | TG | chr8:133973099-133973472 | 373 |  |
| AMPL7155234815 | GATCCATGTCAGGGCATCTGTT | GGTTAAAAGATTAAAGGAGAGTCCATGGA | | TG | chr8:133975090-133975459 | 369 |  |
| AMPL7155234782 | CTCTATCTCTGACACTTTCTCTCTCTGT | ATGACACAAAATTTGGCCTTATCTGTG | | TG | chr8:133978675-133979049 | 374 |  |
| AMPL7155234853 | CTCTGCCCTAACTAGGAGGGAT | TTCTAAAACCACAGAGCCAGCAGAA | | TG | chr8:133979920-133980281 | 361 |  |
| AMPL7155234846 | GCCATCATTTTAGTGAGATTTGAGTTAGG | AGTGCTGGGTATGCTTCTGTTTT | | TG | chr8:133981539-133981913 | 374 |  |
| AMPL7155234778 | TCAAAGCTAAGAATCCACTCATGCA | CCATTTCCTTATCTGTGAACGAGGAT | | TG | chr8:133983869-133984241 | 372 |  |
| AMPL7155234860 | TCCCAAATGTCTGCTGAACAATGTA | CTTGAACCCAATGAGTCTTCATTAATCTAC | | TG | chr8:133984731-133985104 | 373 |  |
| AMPL7155234845 | TGTTTGTTAGCTCAGCATCATCCA | GTTTAATTCATAGGAGCTGGAATTAATGCG | | TG | chr8:133995422-133995794 | 372 |  |
| AMPL7155234791 | TTGCACAGGAATGGAGACCAAGA | GCATCCTAGGTTATCGTAGTGTCTCA | | TG | chr8:134024016-134024390 | 374 |  |
| AMPL7156642723 | ATGCATGAGTGAAGTAACATCTCTGAG | AACTCACCAAGTGTTTAAATAGTCCCTT | | TG | chr8:134025784-134026088 | 304 |  |
| AMPL7155234835 | CATTCAGAATGCCAGTGGAGAGA | TGAGTCCCATTTGGATAGATTAGAATGGA | | TG | chr8:134029945-134030319 | 374 |  |
| AMPL7155234851 | CATTTCCTGAAGCTGCCTAATTTCTG | GAGTGTCCTTAGGCAAATCACCTAA | | TG | chr8:134031706-134032080 | 374 |  |
| AMPL7155234775 | GAGGAGTCCTGTGTCAACCAAG | ATTTCCACTGTGTGCCTAGCTT | | TG | chr8:134034145-134034519 | 374 |  |
| AMPL7155234790 | CTGCCATAGACAGCACCATGAT | AGCCAACTAGACTTTTGCAGCA | | TG | chr8:134041987-134042354 | 367 |  |
| AMPL7155234770 | GCTTGGAGGAAGGATGCAGAAC | GTGACACCTCCTTCCTAGGTCT | | TG | chr8:134107183-134107535 | 352 |  |
| AMPL7155234768 | CATAGATGGATAACCAGTATTGGCATTCA | TCAGCAATTGGTCAATATCTCTTGATCA | | TG | chr8:134108343-134108717 | 374 |  |
| AMPL7155234857 | GCTTCCCAGAGAGAAAATTCTAGAGC | CGTGCCTTTATTTCTCATTCATGACC | | TG | chr8:134125567-134125941 | 374 |  |
| AMPL7155234865 | GGGAATGGGAAGAAGGTGTTCTT | GGTTAAGTGGTGTGTCCCATGT | | TG | chr8:134128708-134129045 | 337 |  |
| AMPL7155234864 | GCCCTAAACAGGATACTTGGATATGA | GATGAGTCTATCGATGCAAATTGGG | | TG | chr8:134143915-134144289 | 374 |  |
| AMPL7155234861 | CAGCCTTGACACCTACAGATACA | GCACAGCAGATGCTGAGATCAA | | TG | chr8:134145589-134145963 | 374 |  |
| AMPL7155234858 | CCCTCTAAAGGGCATTAGCAAGA | TCACACATTAGAGTCAACAATGCTTCA | | TG | chr8:134146794-134147166 | 372 |  |

Fwd, forward; Rev, reverse; a, physical position of the amplicons was obtained from the human assembly (GRCh37/hg19).

Supplementary Table S2: PCR primers used to validate gene sequence variants

| Gene | Variants (nucleotide change) | Primers (5'-3') | Primers (3'-5') | Amplicon size |
| --- | --- | --- | --- | --- |
| DUOX2 | c.227C>T | GGGATGGAGTGCTTAGACAGAG | GCCCTCACCAAAGAAGACCC | 216 |
| DUOX2 | c.1295G>A | GAAGAACCGTAGTGCCAATAG | GGGTTTGGAACAGTATTGCC | 322 |
| DUOX2 | c.1300_1320delCGAGATATGGGGCTGCCCAGC | GAAGAACCGTAGTGCCAATAG | GGGTTTGGAACAGTATTGCC | 322 |
| DUOX2 | c.1304A>G | TGACCTGGGGGTCCACATTAG | TGACCTGGGGGTCCACATTAG | 223 |
| DUOX2 | c.1588A>T | CTCTTTTCTCACCTGGGTCCTTGG | CTCTTTTCTCACCTGGGTCCTTGG | 215 |
| DUOX2 | c.2033A>G | TCGCTTGTGATAATGGAGTCGT | CAAGTTACAGAGTGAGAGGAAAGC | 394 |
| DUOX2 | c.2048G>T | TCGCTTGTGATAATGGAGTCGT | CAAGTTACAGAGTGAGAGGAAAGC | 394 |
| DUOX2 | c.2148+1G>T | CTGGGGACATCTGCTGAACTA | GAGCTCTGTAGCTGAGGATAGG | 339 |
| DUOX2 | c.2335G>A | TGGACTTTGGGGAGATATGAAGG | TGGACTTTGGGGAGATATGAAGG | 289 |
| DUOX2 | c.2654G>A | GGTGGCAGGCTATCCAAG | GAGTTTAGGATGTAGTGTTTAGGC | 307 |
| DUOX2 | c.2716T>C | CTTCTCAGTGCCATTTCCCG | GACACAGAGCTGCGTGAAG | 299 |
| DUOX2 | c.2779A>G | CTTCTCAGTGCCATTTCCCG | GACACAGAGCTGCGTGAAG | 299 |
| DUOX2 | c.3200C>T | GGCGTGTTTGCAGATCGT | CACATAGCGGTTGAGGAAAGTC | 352 |
| DUOX2 | c.3329G>A | CTGTGCCAAGCTGATGTAACCT | GAGTTCCATCTCCCCACTGTTT | 346 |
| DUOX2 | c.3478_3480delCTG | TCCCTCAGGATCGGAGGTAGA | GCCCGGCCTGCCTATTTC | 245 |
| DUOX2 | c.3632G>A | TCTGTTTCTAGCCTTCAGGACAAGC | CCTCTTTCACCTTCCTGTCCCATC | 368 |
| DUOX2 | c.3693+1G>T | TCATGTATGTCTTCGCCTCC | TCATGTATGTCTTCGCCTCC | 209 |
| DUOX2 | c.3967G>A | TCCCTGTCTATGACCTCCAG | GAAGCAGGGTTCCCAGTG | 279 |
| DUOX2 | c.4027C>T | TCCCTGTCTATGACCTCCAG | GAAGCAGGGTTCCCAGTG | 279 |
| DUOX2 | c.4537G>C | CCAAGGAGAGTGAGCACCTTTAG | AAGAGAAGGCAGGATACTGGAAG | 226 |
| DUOX2 | c.4561G>T | CCAAGGAGAGTGAGCACCTTTAG | AAGAGAAGGCAGGATACTGGAAG | 226 |
| DUOXA2 | c.398G>A | TCCTGTCTGAATCCGCTTAGTT | GCTACTCGGTGTGAACTTCTCC | 226 |
| DUOXA2 | c.413dupA | TCCTGTCTGAATCCGCTTAGTT | GCTACTCGGTGTGAACTTCTCC | 226 |
| DUOXA2 | c.738C>G | CTCCTCTCCAACGTGCTGCTCT | ATTGAGAGGGGCAAATAGGGTA | 314 |
| SLC26A4 | c.1286C>A | GACACAAGGGAGAAGGACGA | GACCGACTGCCAAGAGAGAC | 275 |
| TG | c.1514G>A | CCAAAGAGACTCCAGCAAAACC | AGTACCATCCTTCTTAGCAGCTT | 240 |
| TG | c.2060_2060delG | CTTCCAGGCTCAAGAGTCAGAG | ACAGACTTACATTTCTTGGGCTTC | 251 |
| TG | c.2762-1G>A | GGGCACACATGCTTCATGG | CGAGAACTGTTGGAAGCTGAAAC | 166 |
| TG | c.3416C>T | GGAAGGGACACTTTTGACATCTG | CAGCTATGGAAGCAGTAGAGCTG | 300 |
| TG | c.4366G>A | GGGTGACCTCTACCTTATCCTGTG | AGTAGCCTCTCTCCAGTGCTGA | 336 |

Supplementary Table S3: Ion Torrent PGM^TM^ statistics and potential disease variants in patients with CH.

| Patients ID | ≥Q20 bases(%) | Mapped reads | Average base coverage depth | Percent reads on target | Uniformity of base coverage | Coverage≥20× (% bp) | Coverage≥100× (% bp) | Coverage≥500× (% bp) | Number of variants detected | | | |  |
| --- | --- | --- | --- | --- | --- | --- | --- | --- | --- | --- | --- | --- | --- |
|  |  |  |  |  |  |  |  |  | Synonymous | Insertions and deletions | Non-synonymous | intronic | total |
| 1 | 91.91% | 94386 | 445.1 | 98.65% | 91.39% | 98.39% | 93.13% | 45.09% | 9 | 1 | 16 | 50 | 76 |
| 2 | 92.10% | 942537 | 4207 | 98.77% | 87.66% | 100.00% | 99.48% | 94.94% | 4 | 1 | 9 | 42 | 56 |
| 3 | 92.59% | 95461 | 427 | 98.38% | 94.75% | 98.52% | 94.70% | 44.44% | 6 | 2 | 15 | 39 | 62 |
| 4 | 91.15% | 212625 | 1030 | 99.15% | 91.60% | 99.31% | 97.87% | 85.81% | 4 | 0 | 11 | 39 | 54 |
| 5 | 91.65% | 89050 | 371.3 | 98.84% | 68.85% | 97.07% | 69.64% | 28.80% | 7 | 0 | 14 | 40 | 61 |
| 6 | 90.71% | 223640 | 1047 | 99.18% | 93.28% | 99.33% | 97.86% | 84.62% | 5 | 1 | 12 | 39 | 57 |
| 7 | 92.44% | 167830 | 818.4 | 99.03% | 89.32% | 98.61% | 95.92% | 75.90% | 10 | 0 | 11 | 40 | 61 |
| 8 | 91.33% | 183159 | 867.7 | 99.13% | 89.16% | 97.33% | 94.47% | 79.82% | 5 | 1 | 11 | 46 | 63 |
| 9 | 90.29% | 255704 | 1118 | 99.00% | 88.90% | 98.69% | 95.54% | 84.55% | 7 | 0 | 12 | 51 | 70 |
| 10 | 90.47% | 410885 | 1907 | 98.75% | 87.29% | 99.01% | 95.97% | 89.18% | 6 | 1 | 14 | 43 | 64 |
| 11 | 91.97% | 164685 | 758.2 | 99.05% | 87.23% | 97.68% | 93.64% | 70.52% | 6 | 2 | 18 | 78 | 104 |
| 12 | 88.37% | 200425 | 1558 | 99.01% | 90.66% | 98.48% | 95.27% | 82.96% | 6 | 0 | 13 | 45 | 64 |
| 13 | 88.36% | 284598 | 1203 | 98.83% | 90.39% | 99.51% | 97.40% | 86.28% | 5 | 0 | 14 | 52 | 71 |
| 14 | 91.15% | 186412 | 880.8 | 99.06% | 89.42% | 98.06% | 94.78% | 78.94% | 6 | 0 | 14 | 45 | 65 |
| 15 | 90.86% | 398407 | 1890 | 98.97% | 88.53% | 98.99% | 96.62% | 89.99% | 9 | 0 | 16 | 46 | 71 |
| 16 | 92.20% | 156059 | 710.3 | 99.06% | 88.16% | 98.60% | 95.05% | 73.23% | 3 | 0 | 16 | 52 | 71 |
| 17 | 89.32% | 142607 | 649 | 97.75% | 89.35% | 99.04% | 93.88% | 61.92% | 6 | 0 | 12 | 50 | 68 |
| 18 | 92.14% | 133325 | 620.3 | 99.10% | 89.42% | 98.29% | 94.52% | 68.98% | 7 | 0 | 10 | 47 | 64 |
| 19 | 90.78% | 230747 | 993.7 | 98.93% | 91.28% | 98.91% | 97.50% | 85.28% | 6 | 0 | 15 | 47 | 68 |
| 20 | 92.41% | 135687 | 665.5 | 99.07% | 89.55% | 97.40% | 94.85% | 71.66% | 8 | 0 | 12 | 50 | 70 |
| 21 | 88.82% | 498730 | 2216 | 98.73% | 88.23% | 99.84% | 97.39% | 90.27% | 9 | 0 | 11 | 42 | 62 |
| Mean | 91.00% | 247950 | 1161.1 | 98.88% | 88.78% | 98.62% | 94.55% | 74.91% | 6 | 0 | 13 | 47 | 67 |
| Median | 91.15% | 186412 | 880.8 | 99.00% | 89.35% | 98.61% | 95.27% | 79.82% | 6 | 0 | 13 | 46 | 64 |

Q20, 99% certainty that the correct base was called；Mapped reads，total number of reads mapped to the reference genome；Average base coverage depth，the average number of reads of all targeted reference bases，this is the total number of base reads on target divided by the number of targeted bases and therefore includes any bases that had no coverage; Percent reads on target , the percentage of filtered reads mapped to any targeted region relative to all reads mapped to the reference ; Uniformity of base coverage , the percentage of bases in all targeted region (or whole genome)covered by at least 20% of the average base coverage depth reads ; Coverage≥20×, the percentage of targeted bases covered by at least twenty reads ; Coverage≥100×,the percentage of targeted bases covered by at least one hundred reads ; Coverage≥500×, the percentage of targeted bases covered by at least five hundred reads.

Supplementary Table S4: Classification and evidence of 30 variants.

| Gene | Amino Acid change | Classification | PVS1 | PS3 | PS4 | PM1 | PM2 | | | PM3 | PM4 | PP3 | BA1 |
| --- | --- | --- | --- | --- | --- | --- | --- | --- | --- | --- | --- | --- | --- |
|  |  |  | Truncation variants | Functional study | Frequency in normal control | Located in functional domain | 1000 genomes (CHB) | GnomAD  (East Asian) | ExAC (East Asian) | A pathogenic variant detected  *in trans* | Protein length changing variant | *In silico* prediction | MAF is too high for disorder（>0.05） |
| DUOX2 | IVS17+1G>T | P | Yes |  | 0 | NA | / | / | / |  |  | D |  |
| DUOX2 | IVS28+1G>T | P | Yes |  | 0 | NA | 0 | 0.001537 | 0.001502 | Yes |  | D |  |
| DUOXA2 | p.Y138X | P | Yes |  | 0 | NA | / | 0.003699 | 0.002788 | Yes (Yi et al., 2013) |  |  |  |
| DUOXA2 | p.Y246X | P | Yes | Damage (Zamproni et al., 2008) | 0 | NA | / | 0.001880 | 0.002525 |  |  |  |  |
| DUOX2 | p.K530X | P | Yes |  | 0 | Heme pero | 0 | 0.009274 | 0.009244 |  |  |  |  |
| DUOX2 | p.R885Q | P |  | D (Jim et al., 2014) | 0 | EF-2 | 0.0049 | 0.0019 | 0.0015 | Yes (Jiang et al., 2016) |  | D |  |
| DUOX2 | p.R1110Q | P |  | D (Narumi et al., 2011) | 0 | NA | 0.0049 | 0.002597 | 0.0025 |  |  | D |  |
| DUOX2 | p.L1160del | P |  | D (Narumi et al., 2011) | 0 | Helical | / | 0.000371 | 0.0005 | Yes (Narumi et al., 2011) | YES | D |  |
| TG | p.C687LfsX34 | P | Yes |  | 0 | Type 1-6 | / | / | / |  |  |  |  |
| TG | IVS10-1G>A | P | Yes |  | 0 | NA | / | 0 | / |  |  | D |  |
| DUOX2 | p.R434_S440del | LP |  |  | 0 | Heme pero | / | / | / |  | YES | D |  |
| DUOX2 | p.S906P | LP |  |  | 0 | EF-3 | / | / | 0.0000 |  |  |  |  |
| DUOX2 | p.P76L | VUS |  |  | 0 | Heme pero | / | 0.0006 | 0.0005 |  |  |  |  |
| DUOX2 | p.R432H | VUS |  |  | 0 | Heme pero | 0 | 0.0004769 | 0.0007 |  |  | D |  |
| DUOX2 | p.D435G | VUS |  |  | 0 | Heme pero | / | 0.0012 | 0.0001 |  |  |  |  |
| DUOX2 | p.R683L | VUS |  |  | 0 | NA | 0 | 0.0068 | 0.0038 | Yes (Fu et al., 2015) |  | D |  |
| DUOX2 | p.V779M | VUS |  |  | 0.005 | NA | 0.0049 | 0.004094 | 0.0056 |  |  |  |  |
| DUOX2 | p.M927V | VUS |  |  | 0 | EF-3 | / | / | 0.0009 |  |  |  |  |
| DUOX2 | p.R1211H | VUS |  |  | 0.005 | NA | / | 0 | 0.0005 |  |  | D |  |
| DUOX2 | p.A1323T | VUS |  |  | 0 | FAD binding | 0 | / | 0.0007 |  |  |  |  |
| DUOX2 | p.L1343F | VUS |  |  | 0 | FAD binding | 0 | 0.0099 | 0.0068 |  |  |  |  |
| DUOX2 | p.G1521X | VUS |  |  | 0 | NADPH binding | NA | 0.001044 | 0.000809 |  |  |  |  |
| DUOX2 | p.G1513R | VUS |  |  | 0 | NADPH binding | / | / | 0.0000 |  |  | D |  |
| DUOXA2 | p.R133H | VUS |  |  | 0 | NA | / | / | / |  |  |  |  |
| TG | p.G505D | VUS |  |  | 0 | NA | / | / | / |  |  |  |  |
| TG | p.S1139L | VUS |  |  | 0 | Type 1-9 | 0 | 0.0008519 | 0.0007 |  |  |  |  |
| TG | p.G1456R | VUS |  |  | 0 | Type 2 | / | 0.0005301 | 0.0006 |  |  |  |  |
| SLC26A4 | p.A429E | VUS |  |  | 0 | Helical | / | 0.0003712 | 0.0006 |  |  |  |  |
| DUOX2 | p.H678R ^$^ | B |  |  |  | NA | 0.0437 | 0.05001 | 0.0536 |  |  |  | 0.091 |
| DUOX2 | p.S1067L ^$^ | B |  |  |  | NA | 0.0534 | 0.9465 | 0.0525 |  |  |  | 0.085 |

P, pathogenic; LP, likely pathogenic; VUS, variants of uncertain significance; B, benign; D, damaged; T, tolerated; NA, not available; CHB, Han Chinese in Beijing, China; PVS1, null variant (nonsense, frameshift, canonical ±1 or 2 splice sites, initiation codon, single or multi-exons deletion) in a gene where LOF is a known mechanism of disease; PS3, well-established in vitro or in vivo functional studies supportive of a damaging effect on the gene or gene product; PS4, prevalence of the variant in affected individuals is increased compared with controls; PM1, located in a mutational hot spot and/or critical and well-established functional domain; PM2, for recessive disorders, extremely low frequency in 1000 Genomes Project , GnomAD database or Exome Aggregation Consortium (ExAC); PM3, for recessive disorders, detected in trans with a pathogenic variant; PM4, protein length changes as a result of in-frame deletions/insertions in a non-repeat region, or stop-loss variants; PP3, multiple lines of computational evidence support a deleterious effect on the gene or gene product (detailed prediction results shown in Tables 3 and 4); BA1, allele frequency is >5% in 1000 Genomes Project, GnomAD database , Exome Aggregation Consortium (ExAC), or control population.

Supplementary Table S5: Clinical characteristics of DH patients in the validation cohort (n=32) and the validated variants.

| Patient ID | Age, sex | Birth weight(g) | Gestational age (week+day) | Thyroid widths, cm (age) | transient or permanent CH | Screening TSH (uIU/ml) | At confirmative diagnosis, before Tx | | | Gene | Variants | |
| --- | --- | --- | --- | --- | --- | --- | --- | --- | --- | --- | --- | --- |
|  |  |  |  |  |  |  | age | TSH (uIU/ml) | FT4 (ng/dl) |  | Non-polymorphic | polymorphic |
| 22 | 1y5m, M | 3250 | 39+2 | 1.6(30d) |  | >100 | 35d | 100 | 0.1 | DUOX2 | p.R1110Q, IVS28+1G>T |  |
| 23 | 5y6m, F | 3440 | 40+2 | 1.6(25d) | permanent | 11.9 | 25d | 12.44 | 1.15 | DUOX2 | p.R683L, p.L1343F | p.H678R, p.S1067L |
| 24 | 6y11m, F | 4100 | 40+3 | 1.4(54d) | permanent | 33.1 | 54d | 100 | 1.14 |  |  |  |
| 25 | 1y3m, F | 2930 | 38+5 | 1.1(31d) |  | 76.7 | 31d | >100 | 0.27 | DUOX2 | p.K530X, IVS28+1G>T | p.H678R, p.S1067L |
| 26 | 1y2m, F | 2700 | 41+2 | 3.0(45d) |  | 44 | 31d | 860.476 | <0.1 | DUOX2 | p.R1110Q | p.S1067L |
| 27 | 5y1m, F | 3900 | 39 | 1.3(32d) | permanent | 167 | 32d | >100 | 0.51 | DUOXA2 | p.Y246X |  |
| 28 | 6y, F | 3780 | 40 | 1.8(28d) | permanent | 646 | 28d | >100 | 0.1 | DUOX2 |  | p.H678R, p.S1067L |
| 29 | 11m | 4100 | 39 | 1.6(24d) |  | 154 | 24d | >100 | 0.02 | DUOX2 | p.R683L, p.L1343F | p.H678R, p.S1067L |
| 30 | 1y | 2900 | 39+3 | 1.05(41d) |  | 168 | 41d | 25.89 | 0.54 |  |  |  |
| 31 | 9y8m，F | 3400 | 38 | 1.1(57d) | permanent | 54.6 | 57d | >100 | 0.669 |  |  |  |
| 32 | 9m, F | 3300 | 40 | 1.2(15d) |  | 12.3 | 15d | 45.11 | 1.06 |  |  |  |
| 33 | 10m, F | 3000 | 40+3 | 1.08(26d) |  | 13.5 | 26d | 22.5 | 1.25 |  |  |  |
| 34 | 11m, M | 3550 | 39+4 | 2.0(47d) |  | <9 | 47d | 53.56 | 0.63 | DUOX2 | p.R1110Q |  |
| 35 | 10m, M | 3500 | NA | 1.3(41d) |  | 77.8 | 41d | 32.02 | 0.74 |  |  |  |
| 36 | 9m, F | 3300 | 39+4 | 1.1(43d) |  | 16.4 | 43d | 14.54 | 1.36 | DUOX2 |  | p.H678R, p.S1067L |
| 37 | 9m, F | 3500 | 39 | 1.4(30d) |  | 51.3 | 30d | >100 | 0.23 | DUOX2 |  | p.H678R, p.S1067L |
| 38 | 5y4m, M | 3200 | 39+2 | 1.15(30d) | permanent | 12.4 | 30d | 27.61 | 1.26 | DUOX2 | p.V779M |  |
| 39 | 2y4m, M | 2700 | 41+2 | 2.1(38d) |  | 11.9 | 38d | 9.98 | 1.29 | DUOX2 |  | p.H678R, p.S1067L |
| 40 | 3y4m, M | 3100 | 41+2 | 2.6(50d) |  | 80 | 50d | >100 | NA |  |  |  |
| 41 | 4y2m, F | 2900 | 39+4 | 1.8(30d) |  | 9.76 | 30d | 39.11 | 1.12 |  |  |  |
| 42 | 5y, F | 4200 | 42+1 | 1.5(47d) |  | 9.01 | 47d | 18.9 | 1.29 | DUOX2 | p.R1110Q | p.S1067L |
| 43 | 6y10m, F | 2100 | 37 | 1.1(55d) | permanent | 31.2 | 55d | >100 | 0.08 | DUOX2 |  | p.H678R, p.S1067L（Hom, CC) |
| 44 | 5y1m, M | 3600 | 39+6 | 1.2(45d) | permanent | 9.56 | 45d | 73.4 | 1.13 |  |  |  |
| 45 | 7y4m, M | 3755 | 41+3 | 1.07(27d) | permanent | 28.3 | 27d | >100 | 0.48 | DUOX2 | p.R1110Q | p.S1067L |
| 46 | 2y1m, F | 3360 | 40 | 1.3(38d) |  | 96.2 | 38d | 94.55 | 0.6 | DUOX2 |  | p.H678R, p.S1067L |
| 47 | 2y1m, F | 3100 | 40 | 1.4(24d) |  | 44 | 20d | 15.33 | 0.66 | DUOX2 | IVS28+1G>T |  |
| 48 | 4y, F | 2600 | 38 | 1.06(46d) |  | 54.6 | 46d | 56.11 | 0.56 | DUOX2 | p.K530X | p.H678R, p.S1067L |
| 49 | 1y1m, M | 3200 | 39+2 | 1.09(30d) |  | 25.2 | 30d | 10.15 | 0.96 |  |  |  |
| 50 | 1y, F | 3400 | 39+5 | 1.1(25d) |  | 33.4 | 25d | >100 | 0.38 | DUOX2 | p.K530X, p.G1521X | p.H678R, p.S1067L |
| 51 | 4y, M | 3180 | 39 | 1.4(60d) | permanent | 19.5 | 60d | 11.3 | 0.827 |  |  |  |
| 52 | 7y, M | 3100 | 40 | 1.12(24d) |  | 44 | 24d | 15.33 | 1.34 | DUOX2 | IVS28+1G>T |  |
| 53 | 6y9m, M | 3170 | 39 | 1.19(46d) | permanent | 66.8 | 46d | >100 | 0.65 | DUOX2 | p.K530X | p.S1067L(Hom, CC), p.H678R(Hom) |
| Normal |  |  |  |  |  | 0-8 |  | 0-8 | 0.93-1.7 |  |  |  |

m, month; d, day; y, year; F, female; M, male; CH, congenital hypothyroidism; TSH, thyroid-stimulating hormone; FT4, free tetraiodothyronine; Tx: L-thyroxine; Hom, homozygous; NA, data not available.
